# A Comparative Study of the Antihypertensive and Cardioprotective Potentials of Hot and Cold Aqueous Extracts of *Hibiscus sabdariffa* L. in Relation to Their Metabolic Profiles

**DOI:** 10.3389/fphar.2022.840478

**Published:** 2022-02-23

**Authors:** Mohamed A. Salem, Shahira M. Ezzat, Kawkab A. Ahmed, Saleh Alseekh, Alisdair R. Fernie, Reham M. Essam

**Affiliations:** ^1^ Department of Pharmacognosy, Faculty of Pharmacy, Menoufia University, Shibin Elkom, Egypt; ^2^ Max Planck Institute of Molecular Plant Physiology, Potsdam-Golm, Germany; ^3^ Department of Pharmacognosy, Faculty of Pharmacy, Cairo University, Cairo, Egypt; ^4^ Department of Pharmacognosy, Faculty of Pharmacy, October University for Modern Sciences and Arts (MSA), Giza, Egypt; ^5^ Pathology Department, Faculty of Veterinary Medicine, Cairo University, Giza, Egypt; ^6^ Center for Plant Systems Biology and Biotechnology, Plovdiv, Bulgaria; ^7^ Department of Pharmacology and Toxicology, Faculty of Pharmacy, Cairo University, Cairo, Egypt

**Keywords:** ACE inhibition, anthocyanins, metabolomics, organic acids, renin inhibition, extraction temperature, Hibiscus

## Abstract

**Ethnopharmacological relevance:** Since ancient times, *Hibiscus sabdariffa* L. calyces have been used as a folk remedy for the treatment of hypertension. However, it is questionable as to whether there is a difference in the antihypertensive activity of the hot or cold aqueous extracts.

**Aim of the study:** We designed this study to specify the best method for water extraction of the antihypertensive metabolites of *H*. *sabdariffa* and to confirm their *in vivo* antihypertensive capabilities*.*

**Materials and methods:** The powdered dried calyces of *H. sabdariffa* were independently extracted with cold and hot water. A comparative study was performed between the cold and hot aqueous extracts of *H*. *sabdariffa* based on evaluation of the *in vitro* renin and angiotensin-converting enzyme (ACE) inhibition activities. Additionally, both extracts were subjected to an *in vivo* study for the evaluation of their antihypertensive activities in L-N^w^-Nitro arginine methyl ester (L-NAME)–induced hypertensive rats. Further, a metabolomics study was also performed for both extracts to identify their chemical constituents.

**Results:** The cold and hot extracts significantly reduced the angiotensin II, ACE, and aldosterone levels in the plasma. Furthermore, in the myocardium and aorta, decreased iNOS (inducible nitric oxide synthase) levels and elevated eNOS (endothelial nitric oxide synthase), as well as the rise in plasma NO levels, were reported with both extracts, but better results were displayed with the hot extract, leading to a potential antihypertensive effect. Additionally, the cold and hot *Hibiscus* extracts induced a cardioprotective effect through reducing necrosis, inflammation, and vacuolization that results from the induction of hypertension, an effect that was more prominent with the hot extract. Moreover, a comprehensive metabolomics approach using ultra-performance liquid chromatography coupled to tandem mass spectrometry (UPLC–MS/MS) was able to trace the metabolites in each extraction.

**Conclusion:** The extracts showed different anthocyanin and phenolic compounds, but the hot extract showed higher contents of specific phenolics to which the superior antihypertensive and cardioprotective activities could be related.

## Introduction

Hypertension is characterized by persistent high blood pressure (BP), as indicated by 130 mmHg or more for systolic BP or diastolic BP of 80 mmHg or greater according to ACC 2017 guidelines. Hypertension is considered a global health concern with major impact on morbidity and mortality, showing high prevalence in developing countries (20–30%), especially for older generations (Holm et al., 2006). The majority of hypertensive cases are of unknown etiology (primary), while a small percentage could be due to a preexisting condition (secondary) (Yaxley and Thambar, 2015). Several complications could result from uncontrolled hypertension, such as cardiovascular diseases, stroke, and renal failure. Early manifestation of hypertension includes headache, tinnitus, visual changes, and tingling of hands or feet (Holm et al., 2006; Oparil et al., 2018).

Lifestyle modifications (diet, weight, and exercises) are considered the first-line treatment for prehypertensive patients; however, drug therapy should be initiated in partial or non-responsive cases (Mckay et al., 2010). Among the pharmacological drug classes used are diuretics, calcium channel blockers, β-blockers, angiotensin-converting enzyme (ACE) inhibitors, angiotensin II receptor blockers, vasodilators, and centrally acting medications (Jackson and Bellamy, 2015). Although these medications help in controlling the high blood pressure, their side effects include dizziness, headache, dry cough, nausea, and tiredness, which restrict their use, raising the need for alternative treatments (Jackson and Bellamy, 2015). Moreover, many people in developing countries prefer to use herbal medicine due to its affordability and broad safety profile.

*Hibiscus sabdariffa L.* (Roselle) is a plant that belongs to the family Malvaceae which is grown in Egypt, China, Mexico, and Sudan (Da-Costa-Rocha et al., 2014). It is widely used in the food industry and in medicine. *Hibiscus* was found to have a wide range of therapeutic activities including antihypertensive, anticancer, antispasmodic, antifungal, antibacterial, anti-inflammatory, antipyretic, and hepatoprotective effects (Abat et al., 2017). The medicinal effects of *Hibiscus* are attributed to the presence of several phytochemical constituents including the following: minerals, vitamins, anthocyanins, flavonoids, polyphenolic acids, and organic acids (Riaz and Chopra, 2018). The antihypertensive activity of *Hibiscus* extracts is mediated through three main mechanisms: ACE inhibition, vasodilation, and diuretic effects (Ojeda et al., 2010). In traditional medicine, either maceration with cold water or hot decoction is the most widely applied extraction methods for the preparation of *Hibiscus* herbal teas (Rasheed et al., 2018).

In Egypt, *Hibiscus* is a reputed beverage for controlling elevated blood pressure. However, it is believed that the preparation method influences the *Hibiscus* antihypertensive activity. Some people believe that the reduction in blood pressure is achieved only by the cold *Hibiscus* extract, while the hot preparation raises it, although these beliefs were not deeply investigated on a scientific basis. Figuring out the mystery of whether the hot or cold extract possesses antihypertensive potential is essential. Thus, we present herein a comparative metabolomics analysis of cold and hot aqueous extracts of *Hibiscus sabdariffa* L. with respect to their antihypertensive potentials in light of continuing our work on the best extraction method for *Hibiscus* antihypertensive metabolites (Salem et al., 2020). To accomplish such a goal, an *in vitro* assay was initially performed to evaluate the inhibitory activity of *Hibiscus* metabolites on ACE and renin activities. Moreover, LC/MS analysis coupled to multivariate data analysis (MVDA) was performed in order to reveal the different patterns of both extraction methods, highlighting the impact of thermal treatment on chemical composition. Furthermore, an *in vivo* experiment was designed to compare the antihypertensive and the cardioprotective potentials of both extraction methods to come to a conclusion as to whether hot extraction affects the antihypertensive and the cardioprotective capabilities of *Hibiscus* tea or not.

## Materials and Methods

### Plant Materials and Chemicals

*Hibiscus sabdariffa* L. was grown at the experimental station for medicinal and aromatic plants of the Faculty of Pharmacy, Cairo University, Egypt, during the summer of 2018. A voucher specimen (08-06-2018) was kept in the herbarium of Pharmacognosy Department, Faculty of Pharmacy, Cairo University, Cairo, Egypt. The flowers were harvested, and calyces were separated before they were subjected to air drying.

ACE, Histidine-L-hippuryl- L-leucine-chloride (HHL), methanol, sodium borate buffer, sodium hydroxide, renin enzyme, assay buffer (Tris-HCl and sodium chloride), renin substrate (Arg-Glu (EDANS)-Ile-His-Pro-Phe-His-Leu-Val-Ile-His-Thr-Lys(Dabcyl)-Arg), dimethyl sulfoxide (DMSO) solution, aliskiren, methanol, formalin, xylene, alcohol, paraffin, eosin, hematoxylin, paraffin, 3-amino-propyl trioxysilane, hydrogen peroxide, citrate buffer, PBS (Phosphate-buffered saline), 3,3 diaminobenzidine, Mayer’s hematoxylin, and mouse PAP (peroxidase–anti-peroxidase) complex were purchased from Sigma-Aldrich (St Louis, MO, United States). Captopril was purchased from the Egyptian International Pharmaceutical Industries Company (EIPICO, Egypt), while Nω-Nitro-L-arginine methyl ester (L-NAME) was obtained from Alfa Aesar (Lancashire, United Kingdom).

### Extraction of *Hibiscus sabdariffa* L

A pool of 50 g of the dried *Hibiscus* calyces were powdered and divided into two equal parts, each of 25 g, which were subsequently subjected to cold and hot extraction (Ramirez-Rodrigues et al., 2011). For cold maceration, 25 g of the dried fine-powdered calyces were extracted with 500 ml of cold distilled water for 4 h. The decoction was prepared by adding 500 ml of boiling distilled water to 25 g of the dried fine-powdered calyces, and the water temperature was maintained for 15 min. Extracts were, in each case, filtered, and the filtrates were evaporated to dryness by rotary evaporation under reduced pressure, yielding 10.19 g of the cold aqueous extract and 11.06 g of the hot aqueous extract, respectively. The samples were kept at −20°C until further analysis.

### Angiotensin-Converting Enzyme Inhibition Assay

The ACE inhibition potential of the extracts was tested according to a published method (Balasuriya and Rupasinghe, 2012), with minor modifications. In brief, both hot and cold aqueous extracts were dissolved in methanol (100 mg/ml). Serial dilutions were prepared for each test solution (0.1–100 mg/ml) and for each fraction (0.01–100 mg/ml). Captopril, in a concentration range of 0.0001–0.1 mg/ml, was used as the reference drug. The enzyme solution (40 μL, 2-mU ACE prepared in 0.1-M Na borate buffer) was added to 20 μL of each tested dilution of each sample and then incubated at 37°C for 10 min; 40 μL of the HHL substrate (0.8 mM/L) was added to prepare the test solutions. The test solutions were all incubated at 37 C for 1 h, and in order to stop the reaction, 60 μL of 0.5 M sodium hydroxide was added. The buffer solution was added instead of the enzyme solution in order to prepare the blank solution for each sample. Instead of the sample, methanol was used to prepare control solutions. Triplicates were run for each sample. Experiments were carried out in 96-well microplates. Fluorescence was measured at excitation (360 nm) and emission wavelengths (500 nm). The following equation was used to calculate percentages of inhibition (% I ± SD): % Inhibition = Fl. Control- (Fl sample–Fl blank)/Fl control × 100, where Fl. Control: fluorescence of the test solution containing the solvent instead of the extract; Fl. Blank: fluorescence of the test solution containing all the reagents except the enzyme. The IC_50_ ± SD (*n* = 3) values were calculated in mg/mL by linear interpolation.

### Renin Inhibition Assay

The renin inhibitory activity of the extracts was determined according to the previously described method of Bhullar et al. (2014), with some modifications (Bhullar et al., 2014). The renin inhibitory activity of both aqueous extracts was carried out as 50 μL of the renin enzyme was dissolved in 50-mM Tris-HCl buffer (pH 8.0) and 100-mM NaCl (assay buffer) and stored at –80°C for further analysis. DMSO was used to dilute the renin substrate, Arg-Glu (EDANS)-Ile-His-Pro- Phe-His-Leu-Val-Ile-His-Thr-Lys(Dabcyl)-Arg), to reach a concentration of 500 μM. For the experiment, 96-well microplates were used to perform the assay. 20 μL of substrate, 150 μL of assay buffer, and 10 μL of each sample were used to prepare test extracts (10 mg/ml in methanol). Aliskiren, in a concentration of 0.1 mg/ml, was used as a reference drug. 20 μL of substrate, 160 μL of assay buffer and 10 μL of the sample were used to prepare blank samples. 20 μL of substrate, 150 μL of assay buffer, and 10 μL of methanol were used to prepare the positive control samples. For the assay, 10 μL of the renin solution was added to the positive control and test solutions to catalyze the reaction. The reaction mixture was then incubated at 37°C for 45 min. A FLUOstar OPTIMA plate reader was used to measure the fluorescence produced at the excitation wavelength of 340 nm and emission wavelength of 490 nm.

### *In Vivo* Evaluation of the Antihypertensive and Cardioprotective Activities

#### Animals

Male Wistar rats (250–350 g) were purchased from the Modern Veterinary Office for Laboratory Animals, Giza, Egypt, and housed in plastic cages at the animal house of the Faculty of Pharmacy, Cairo University. Animals were allowed free access to standard diet and water *ad libitum*. Rats were kept under constant temperature (23 ± 2°C), constant humidity, and a 12-hour light/dark cycle during the experimental period. All procedures were reviewed and approved by the Ethical Committee for experimentation with laboratory animals, Faculty of Pharmacy, Cairo University, registration number (PT: 2856), following the European Economic Community regulations revised guidelines (86/609/EEC).

#### Induction of Hypertension

Induction of hypertension was performed by oral administration of L-NAME (40 mg/kg) dissolved in normal saline for 4 weeks (Bernátová et al., 1999).

#### Experimental Design

Animals were allocated into seven groups, five rats per group as follows:

**Group 1** (the normal group): rats received normal saline during the whole experimental period (eight weeks).

**Group 2** (the cold *Hibiscus* control group): rats were given the cold aqueous extract (250 mg/kg/day) orally, dissolved in distilled water, during the whole experimental period.

**Group 3** (the hot *Hibiscus* control group): rats were given the hot aqueous extract (250 mg/kg/day) orally, dissolved in distilled water, during the whole experimental period.

**Group 4** (the positive control group): rats were administered L-NAME at a dose of 40 mg/kg orally for 4 weeks. During this period, the systolic blood pressure was measured weekly. After four weeks of administration, the rats that showed definite hypertension (SBP>140 mmHg) were considered hypertensive.

**Group 5** (the standard group): animals received L-NAME (40 mg/kg, p.o.) during the experimental period where captopril (30 mg/kg, p.o.) dissolved in carboxymethylcellulose (CMC) (Sawant and Bodhankar, 2016) was introduced starting from the 4^th^ week up to the 8^th^ week.

**Group 6** (the cold *Hibiscus* extract treatment group): rats were given L-NAME (40 mg/kg, p.o.) during the experimental period, and the cold aqueous extract (250 mg/kg, p.o.) was introduced from the 4^th^ week up to the 8^th^ week.

**Group 7** (the hot *Hibiscus* extract treatment group): rats received L-NAME (40 mg/kg, p.o.) during the experimental period, and the hot aqueous extract (250 mg/kg, p.o.) was introduced from the 4^th^ week up to the 8^th^ week.

Systolic blood pressure (SBP) was measured non-invasively using the tail cuff method through the PowerLab controller device model IN 125/R & MLT125 pulse/pressure transducer (AD instruments, Australia). At the end of the experimental period, blood samples were collected in heparinized tubes from the retro-orbital sinus, and then, animals were sacrificed by cervical dislocation under light anesthesia. Hearts and aorta were carefully and rapidly excised, washed, with ice-cold normal saline, and dried with filter papers. Parts of the heart and aorta from each group were fixed in 10% neutral-buffered formalin for histological and immunohistochemical examinations whereas other parts were frozen at −80°C for biochemical analysis.

#### Histopathological Examination

Sections of 5 μm thickness of the heart and aorta were prepared and stained with hematoxylin and eosin (H&E) for histopathological examination by using a light microscope (Suvarna et al., 2019). Five fields from the microscope were investigated per section/rat (*n* = 5), and grading (0–4) was used to describe the histopathological alterations in the aorta and heart as follows: score (0) indicated the absence of histopathological lesions, score (1) indicated limited focal distribution of lesions, scores (2 and 3) indicated moderate severity with several histopathological lesions, and score (4) indicated severe lesions on the overall examined sections (Dong et al., 1992).

#### iNOS and eNOS Immunohistochemistry

Immunohistochemical detection of inducible and endothelial nitric oxide synthases (iNOS and eNOS) was performed on myocardial and aortic paraffin tissue sections and mounted on 3-amino-propyl-trioxysilane–coated glass slides. The PAP technique was used to perform immunostaining for iNOS and eNOS. In order to quench endogenous peroxidase, slides were incubated in 0.3% H_2_O_2_, then boiled in citrate buffer for 15 min, rinsed with PBS, and processed for staining. Using the monoclonal antibody for iNOS (Cat # sc-7271; dilution:1:100, Santa Cruz Biotechnology Inc., Dallas, TX, United States) and eNOS (Cat # sc-376751; dilution: 1:100 Santa Cruz Biotechnology Inc., Dallas, TX, United States), slides were incubated at 4°C overnight after being preincubated with normal rabbit serum. After PBS wash and using the rabbit-anti-rat serum, sections were incubated, washed, and incubated again using the rat PAP complex (transduction). In the dark, using 0.025% of 3,3 diaminobenzidine, the chromogen reaction was allowed to start. After counterstaining of the slides with Mayer’s hematoxylin, they were examined using a light microscope (Das et al., 2005)**.** The intensity and distribution of staining were graded as negative (no staining), weak, moderate, or strong. By measuring the area % expression from five fields chosen randomly in each section, the quantification of iNOS and eNOS was estimated and averaged by image analysis software (ImageJ, version 1.46a, NIH, Bethesda, MD, United States).

#### Biochemical Parameters

##### Angiotensin I-Converting Enzyme Activity Assay Kit

The activity of the ACE was determined in the prepared plasma using a special ACE activity kit obtained from BioVision incorporated (Cat. #K227-100, California, United States). The assay steps were carried out according to manufacturer’s instructions, and results were expressed as pmol/min/ml.

##### Enzyme-Linked Immunosorbent Assays of Rat Angiotensin II and Aldosterone Levels

The plasma prepared was used to measure angiotensin II and aldosterone using the corresponding rat ELISA kits (Cusabio, Wuhan, China; Cat. # CSB-E04494r; MY BioSource, San Diego, California, United States; Cat.# MBS033119, respectively). Procedures were performed according to manufacturer’s instructions, and results were expressed as pg/ml.

##### Nitric Oxide (NO) Assay Kit

NO was measured in the separated plasma according to the procedures reported in the NO assay kit purchased from Biodiagnostic and research agents (Cat. # NO 25 33, Giza, Egypt). The results were expressed as mmol/ml.

### Analysis of Metabolites by UPLC Coupled to MS

The extracts from each extraction method (10 mg) were reconstituted in 1 ml of 50% aqueous methanol supplemented with 0.25 μg/ml of the internal standard mix (ampicillin and corticosterone). The samples were kept on an orbital shaker for 5 min at 100 rpm before they were centrifuged at 10,000 rpm for 5 min. The supernatants were transferred to LC glass vials, and 2 µL was injected to a high-strength silica (HSS)-C_18_ column (100 mm × 2.1 mm containing 1.7 μm diameter particles, Waters) and was used to separate the metabolites of both extracts using a Waters Acquity UPLC system (Salem et al., 2017). The mass spectra were acquired in the positive and negative ionization mode using a heated electrospray ionization (HESI) source in combination with an Orbitrap-type HRMS (Salem et al., 2016). Metabolites were identified by their mass spectra and comparison with our in-house database and the reference literature reports (Salem et al., 2020). All the obtained data were analyzed and correlated using MetaboAnalyst (Pang et al., 2020).

### Statistical Analysis

Data were expressed as means ± standard error (SEM). Analysis of the results was performed using the one-way analysis of variance test (ANOVA) followed by Tukey’s *post hoc* multiple comparison test. However, for histopathological scoring, results were analyzed using the Kruskal–Wallis ANOVA test, followed by Dunn’s multiple comparison test. For all statistical tests, the level of significance was set at *p < 0.05, **p < 0.01, ***p < 0.001, ****p < 0.0001, and ns: no significance. GraphPadPrism^®^ software package, version 9 (GraphPad Software, Inc., United States) was used to carry out all statistical tests.

## Results

### *In Vitro* Angiotensin-Converting Enzyme and Renin Inhibition Assays

The hot aqueous *Hibiscus* extract showed higher ACE inhibition (IC_50_ = 0.98 ± 0.06 μg/ml) than the cold extract (2.09 ± 0.07 μg/ml), with respect to the standard captopril (IC_50_ = 0.210 ± 0.005 μg/ml). Additionally, both hot and cold extracts showed significant renin inhibition with IC_50_ = 0.371 ± 0.090 and 0.296 ± 0.10 μg/ml for hot and cold extracts, respectively, with respect to the standard aliskiren (IC_50_ = 0.030 ± 0.001 μg/ml).

### *In Vivo* Antihypertensive and Cardioprotective Capabilities

#### Effect of Cold and Hot *Hibiscus* Extracts on Systolic Blood Pressure and Electrocardiographic Parameters in L-NAME–Induced Hypertensive Rats

**FIGURE 1:**
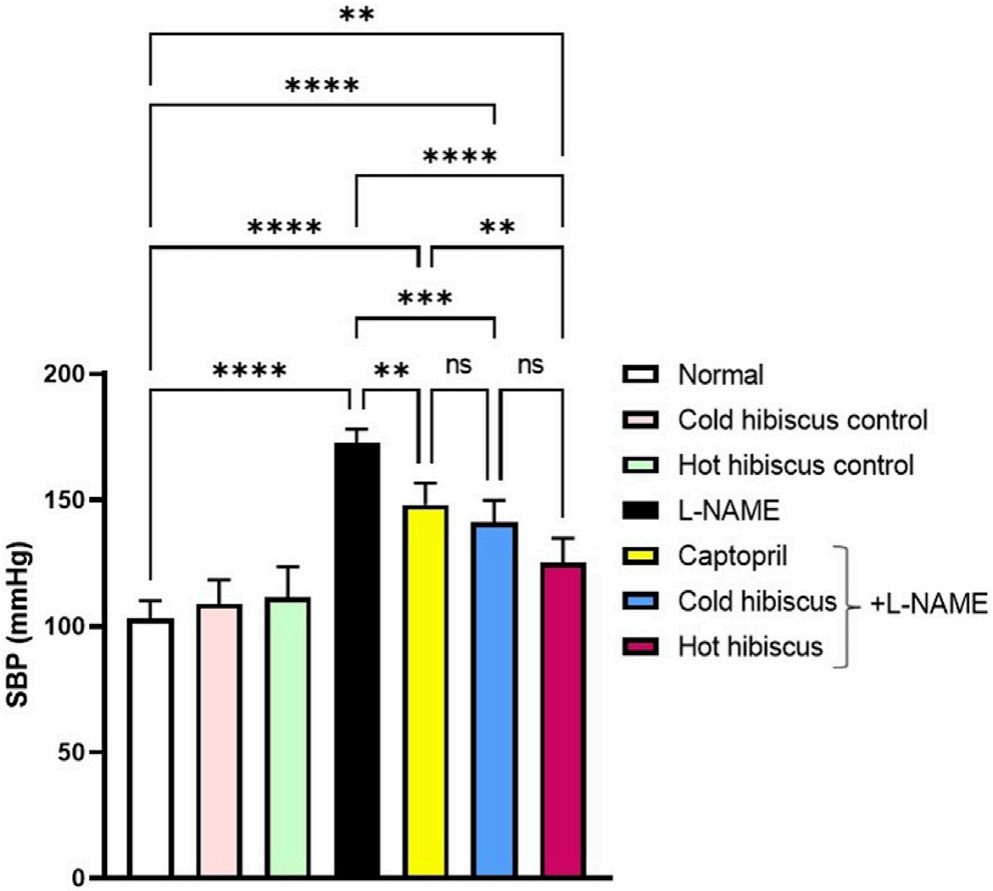
Effect of cold and hot *Hibiscus* extracts of the SBP in L-NAME–induced hypertensive rats. Hypertension was induced by oral administration of L-NAME (40 mg/kg/day) for 4 weeks. Captopril (30 mg/kg) and cold or hot *Hibiscus* extract (250 mg/kg/day) were given orally for further 4 weeks during which L-NAME administration was continued. Results were expressed as means ± SEM (*n* = 5). Statistical analysis was conducted using one-way ANOVA followed by Tukey’s *post hoc* test *p< 0.05,**p< 0.0001, ns: no significance.

Rats receiving L-NAME showed significant increase in the SBP (172.6 ± 2.42 mmHg) compared to the normal group (103.2 ± 3.05 mmHg). However, the captopril group showed significant reduction in the SBP (148.0 ± 3.88 mmHg) compared to the L-NAME group. Moreover, both cold and hot aqueous extract–treated groups showed significant reduction in the SBP (141.0 ± 3.96 and 125.2 ± 4.30 mmHg, respectively) compared to the L-NAME group. The magnitude of reducing the SBP by the hot aqueous extract (****p < 0.0001) was more prominent to that obtained on administration of the cold extract (***p < 0.001) (Figure 1). Nevertheless, no significant changes were detected in the heart rate, R-R interval, QRS interval, and QTc interval, among all groups (Supplementary Figure S1).

#### Effect of Cold and Hot *Hibiscus* Extracts on Angiotensin-Converting Enzyme Activity, Angiotensin II, and Aldosterone Levels

**FIGURE 2:**
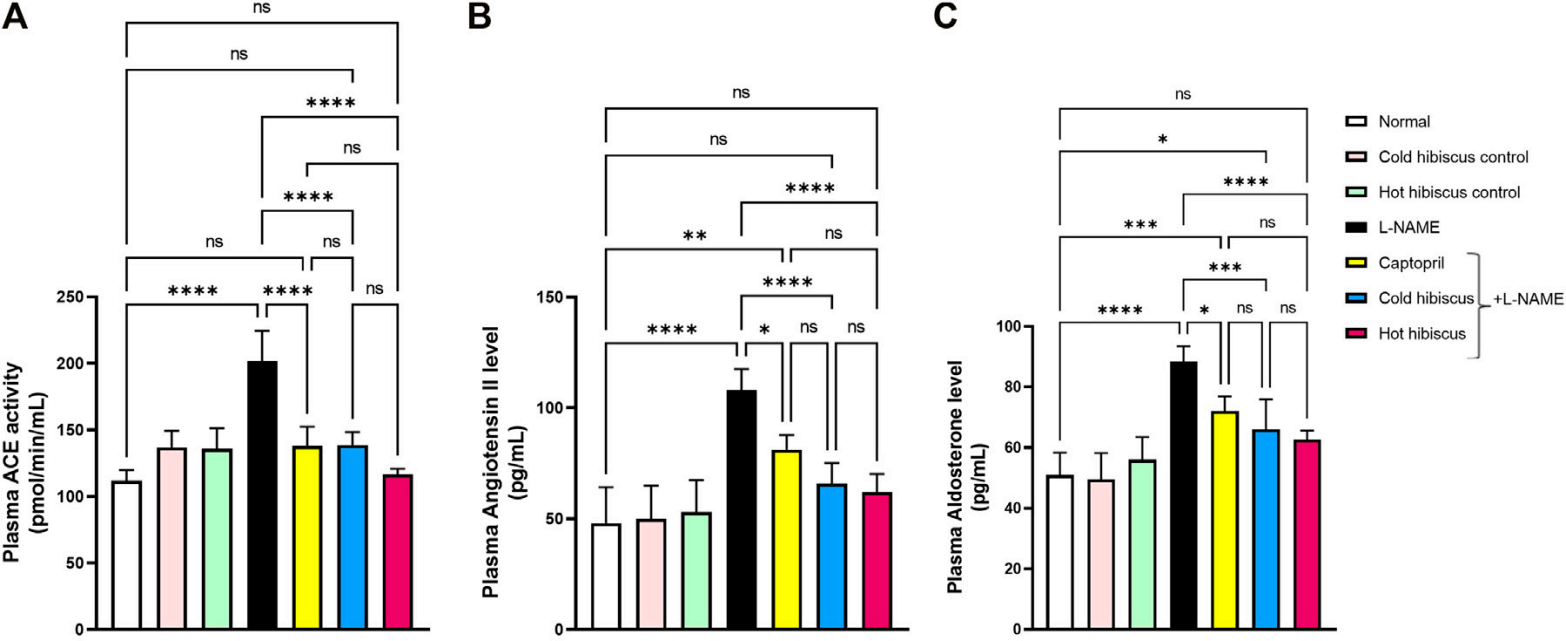
Effect of cold and hot *Hibiscus* extracts on the plasma ACE activity **(A)** angiotensin II **(B)** and aldosterone levels **(C)** in L- NAME–induced hypertensive rats. Hypertension was induced by oral administration of L-NAME (40 mg/kg/day) for 4 weeks. Captopril (30 mg/kg) and cold or hot hibiscus extract (250 mg/kg/day) were given orally for further 4 weeks during which L-NAME administration was continued. Results were expressed as means ± SEM (*n* = 5). Statistical analysis was conducted using one-way ANOVA followed by Tukey’s *post hoc* test *p < 0.05,**p < 0.01,***p < 0.01,****p < 0.0001, ns: no significance.

Plasma ACE activity, angiotensin II, and aldosterone levels were significantly elevated after L-NAME induction when compared to the normal group. There were no significant differences among control rat groups of cold and hot *Hibiscus* extracts. Co-administration of captopril induced marked reduction in the plasma ACE activity (138 ± 6.44 pmol/ml), plasma angiotensin II (81.22 ± 2.95 pg/ml), and plasma aldosterone level (72.05 ± 2.18 pg/ml), when compared to the L-NAME group. Moreover, cold and hot *Hibiscus* extracts normalized the elevated plasma ACE activity and angiotensin II, while only the hot extract normalized the aldosterone level, and the cold extract significantly reduced its level when compared to L-NAME hypertensive rats (Figure 2).

#### Effect of Cold and Hot *Hibiscus* Extracts on Myocardial and Aortic Histopathological Changes in L-NAME–Induced Hypertensive Rats

**FIGURE 3:**
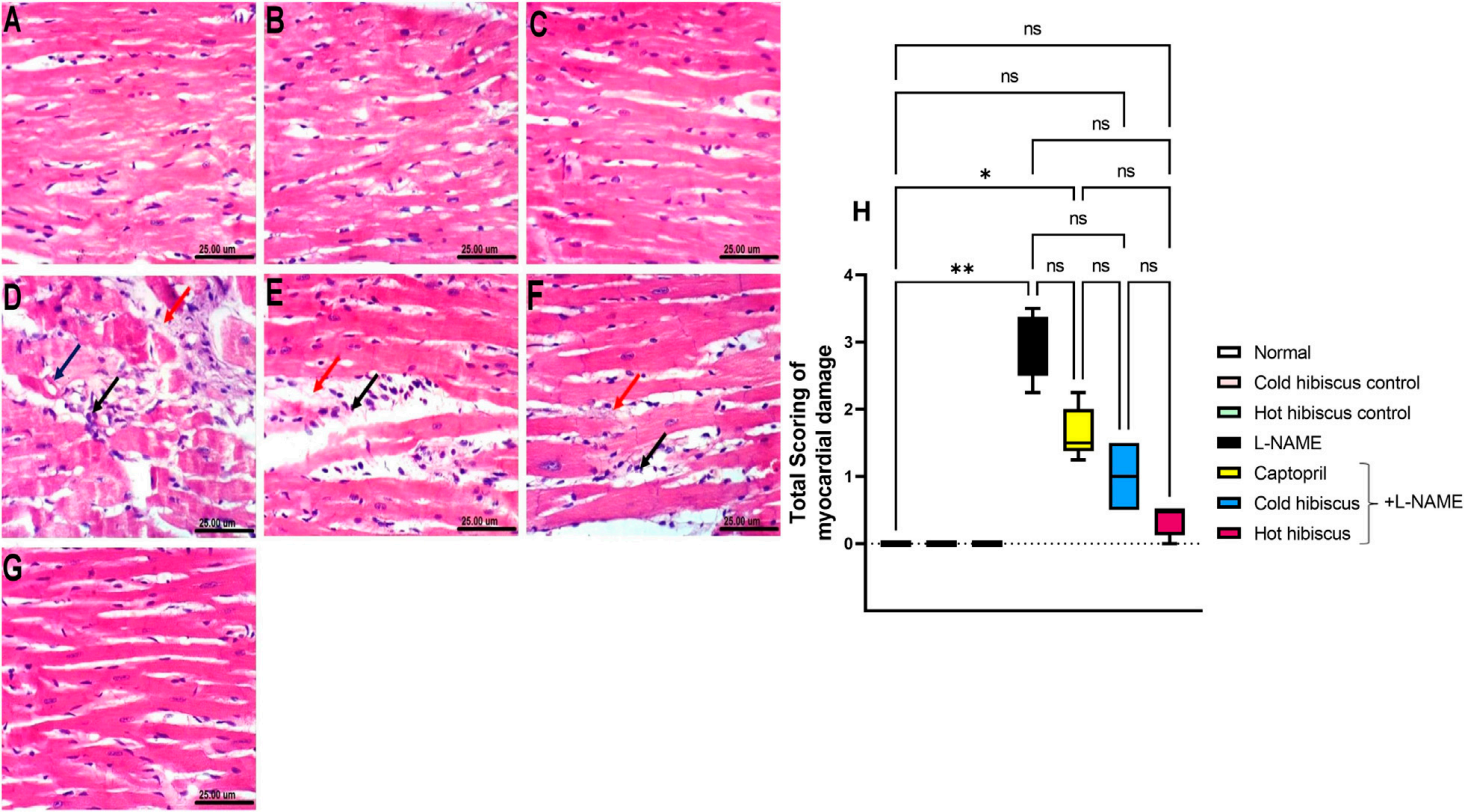
Effect of cold and hot *Hibiscus* extracts on myocardial histopathological changes in L-NAME–induced hypertensive rats. Photomicrograph of H&E-stained heart tissue section (scale bar, 25um): **(A)** normal, **(B,C)** cold *Hibiscus* and hot *Hibiscus* control rats, respectively, showing no histopathological alterations. **(D)** L-NAME–treated rats showing vacuolization of the sarcoplasm of cardiomyocytes (blue arrow) focal cardiomyocytes necrosis associated with mononuclear cell infiltration (black arrow) and intermyocardial edema (red arrow). **(E)** Captopril+L-NAME-treated rats showing mononuclear cell infiltration between cardiomyocytes(black arrow) and intermyocardial edema (red arrow ) **(F)** cold *Hibiscus*+ L-NAME-treated rats showing infiltration of few intermuscular cells (black arrow) with slight edema (red arrow). **(G)** hot *Hibiscus*+ L-NAME-treated rats showing normal histological architecture of myocytes. **(H)** Total histological scoring of myocardial damage. Results were expressed as median and range (*n* = 5). Statistical analysis was conducted using Kruskal–Wallis ANOVA followed by Dunn’s multiple comparison test. *p < 0.05, **p < 0.01,*** *p* <0.001**** *p* <0.0001, ns: no significance.

The myocardium of the normal group and the cold and hot extract control groups showed no histopathological variations in the heart (Figures 3A–C). Conversely, the heart of the L-NAME group showed severe damage described by high vacuolization of the sarcoplasm of cardiac myocytes, in addition to showing focal necrosis of cardiomyocytes associated with mononuclear cell infiltration and intermyocardial edema that was reflected on the histology score (Figures 3D,H). Nevertheless, the heart of the group treated with captopril showed moderate amelioration of the histopathological variations, revealing mononuclear cell infiltration between cardiomyocytes and intermyocardial edema together with deterioration in the total score (Figures 3E,H). However, the cold *Hibiscus* + L-NAME group showed regression of the damage with the infiltration of few intermuscular mononuclear cells and slight edema and a minor change in the myocardial score (Figures 3F,H), while restoration of the normal histological structure of myocytes and scoring of the damage were examined in the heart treated with the hot *Hibiscus* extract (Figures 3G,H).

**FIGURE 4:**
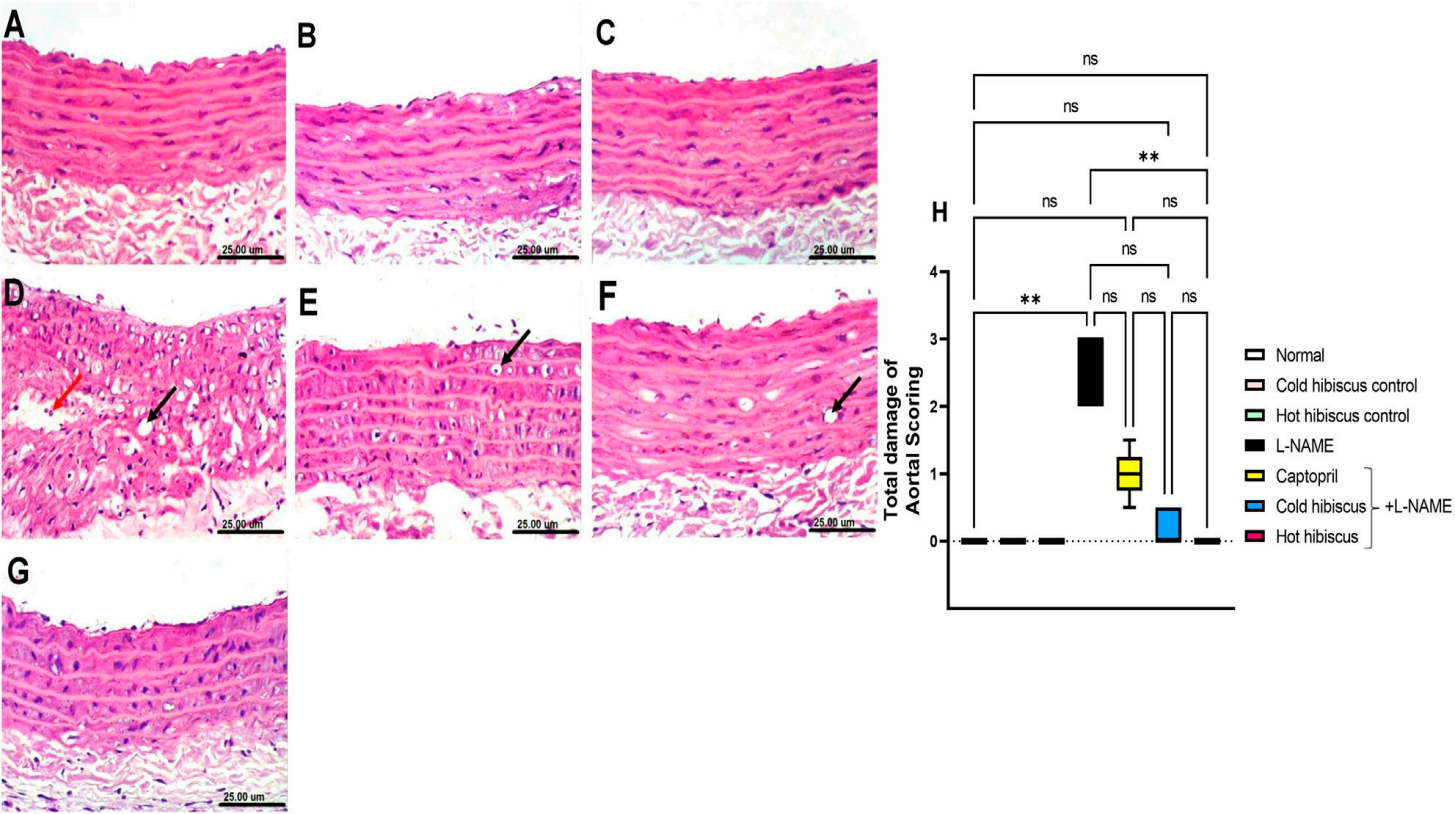
Effect of cold and hot *Hibiscus* extracts on aortic histopathological changes in L-NAME–induced hypertensive rats. Photomicrograph of H&E-stained heart aortic sections(scale bar, 25um): **(A)** normal rat showing normal histological layers. **(B,C)** cold *Hibiscus* and hot *Hibiscus* control rats, respectively, showing no histopathological alterations. **(D)** L-NAME–treated rats showing marked vacuolization of the cells of the tunica media (black arrow) and infiltration of few inflammatory cells (red arrow) **(E)** Captopril+L-NAME-treated rats showing moderate vacuolization of cells of the tunica media (arrow) **(F)** cold *Hibiscus*+ L-NAME showing slight vacuolization of sporadic cells of the tunica media (arrow). **(G)** hot *Hibiscus*+ L-NAME-treated rats showing no histopathological alteration and restoring the normal histology of the aorta **(H)** Total histological scoring of aortic damage. Results were expressed as median and range (*n* = 5). Statistical analysis was conducted using Kruskal–Wallis ANOVA followed by Dunn’s multiple comparison test. *p < 0.05, **p < 0.01,***p < 0.001****p < 0.0001, ns: no significance.

Regarding the aorta, normal and extract groups showed no histopathological variations (Figures 4A–C). In contrast, the aorta section of the L-NAME–treated group showed high vacuolization of cells of the tunica media and infiltration of few inflammatory cells (Figure 4D). These changes were confirmed with the abnormal histological score shown in Figure 4H. However, the aorta section of the group treated with captopril showed moderate vacuolization of cells of the tunica media (Figure 4E). Moreover, restoration of the aorta section was recorded with the group treated with cold *Hibiscus* + L-NAME with slight vacuolization of sporadic cells of the tunica media (Figure 4F). Meanwhile, restoring the normal histology of the aorta was seen in the group treated with the hot *Hibiscus* extract and the total histology score that was significantly depressed when compared to the L-NAME group (Figures 4G,H). Hence, the hot extract showed better restoration in aortic histology than the cold extract.

#### Effect of Cold and Hot *Hibiscus* Extracts on Myocardial and Aortic iNOS and eNOS Immunostaining in L-NAME–Induced Hypertensive Rats

**FIGURE 5:**
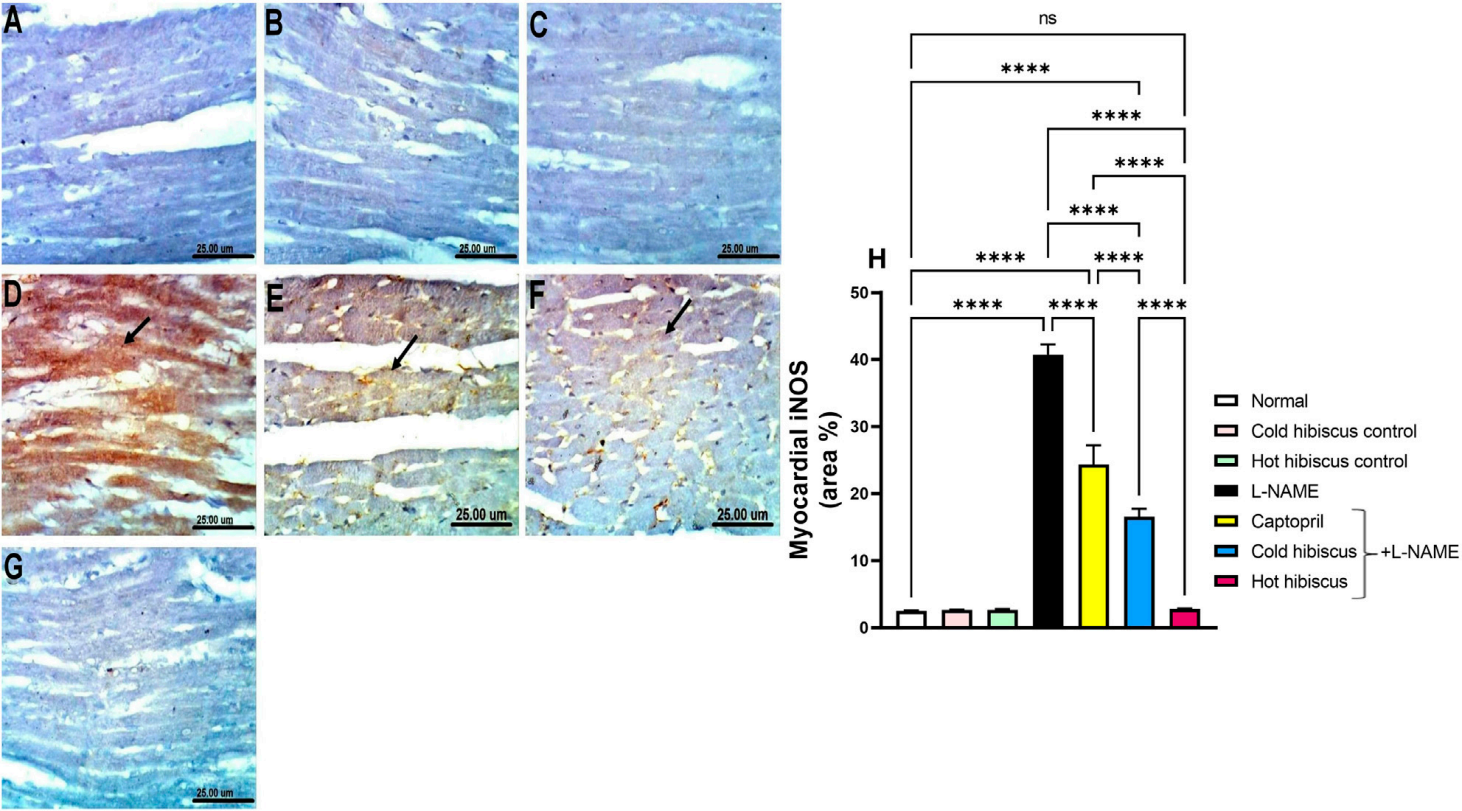
Effect of cold and hot *Hibiscus* extracts on aortic iNOS immunostaining in L-NAME–induced hypertensive rats: Immunohistochemical analysis of the iNOS expression in the aorta of different experimental groups (Scale bar:25 um): **(A)** normal rat, **(B,C)** cold *Hibiscus* and hot *Hibiscus* control rats, respectively, showing no iNOS expression. **(D)** L-NAME–treated rats showing intense iNOS expression (arrow) **(E)** Captopril +L-NAME-treated rats showing moderate iNOS expression (arrow). **(F)** cold *Hibiscus* + L-NAME-treated rats showing moderate to weak iNOS expression (arrow) **(G)** hot *Hibiscus*+ L-NAME-treated rats showing no iNOS expression. **(H)** iNOS immunohistochemical staining area %. Results were expressed as means ± SEM (*n* = 5). Statistical analysis was conducted using one-way ANOVA followed by Tukey’s *post hoc* test *p < 0.05, **p < 0.01, ***p < 0.01, ****p < 0.0001, ns: no significance.

**FIGURE 6:**
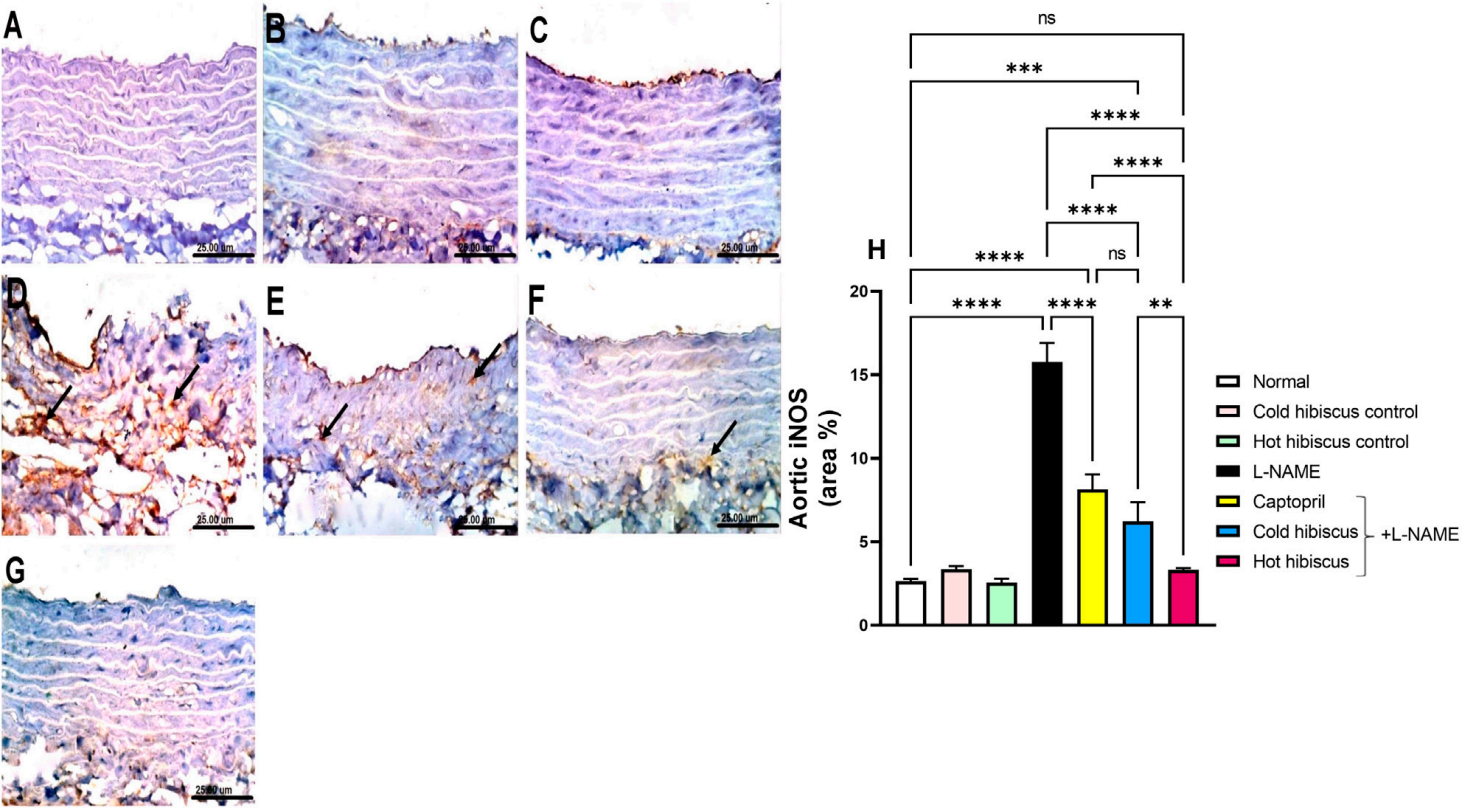
Effect of cold and hot *Hibiscus* extracts on aortic iNOS immunostaining in L-NAME–induced hypertensive rats. Immunohistochemical analysis of iNOS expression in the aorta of different experimental groups (Scale bar:25 um): **(A)** normal rat, **(B,C)** cold *Hibiscus* and hot *Hibiscus* control rats, respectively, showing no iNOS expression. **(D)** L-NAME–treated rats showing intense iNOS expression(arrow) **(E)** Captopril +L-NAME-treated showing moderate iNOS expression (arrow). **(F)** cold *Hibiscus* + L-NAME-treated rats showing moderate to weak iNOS expression (arrow) **(G)** hot *Hibiscus*+ L-NAME-treated rats showing no iNOS expression. **(H)** iNOS immunohistochemical staining area %. Results were expressed as means ± SEM (*n* = 5). Statistical analysis was conducted using one-way ANOVA followed by Tukey’s *post hoc* test *p < 0.05, **p < 0.01, ***p < 0.01,****p < 0.0001, ns: no significance.

Immunohistochemical staining of iNOS in the heart and aorta revealed no expression in the sections of normal rats and in cold or hot *Hibiscus* control rats (Figures 5A–C, Figures 6A–C). On the contrary, an intense positive immune reaction was observed in cardiomyocytes and the aorta of rats treated with L-NAME (Figure 5D, Figure 6D). Sections from rats treated with captopril + L-NAME revealed a moderate iNOS expression (Figure 5E, Figure 6E). Moreover, a weak iNOS immune reaction was recorded in sections of rats treated with the cold *Hibiscus* extract (Figure 5F, Figure 6F). Meanwhile, heart and aorta sections of rats treated with hot *Hibiscus* + L-NAME revealed no iNOS expression (Figure 5G, Figure 6G). The previously mentioned results were confirmed by quantitative estimation of cardiac and aortic iNOS immunostaining in different groups, as shown in Figure 5H, Figure 6H, which showed a significant difference between the cold and hot extracts, granting the hot extract more power in the amelioration of iNOS.

**FIGURE 7:**
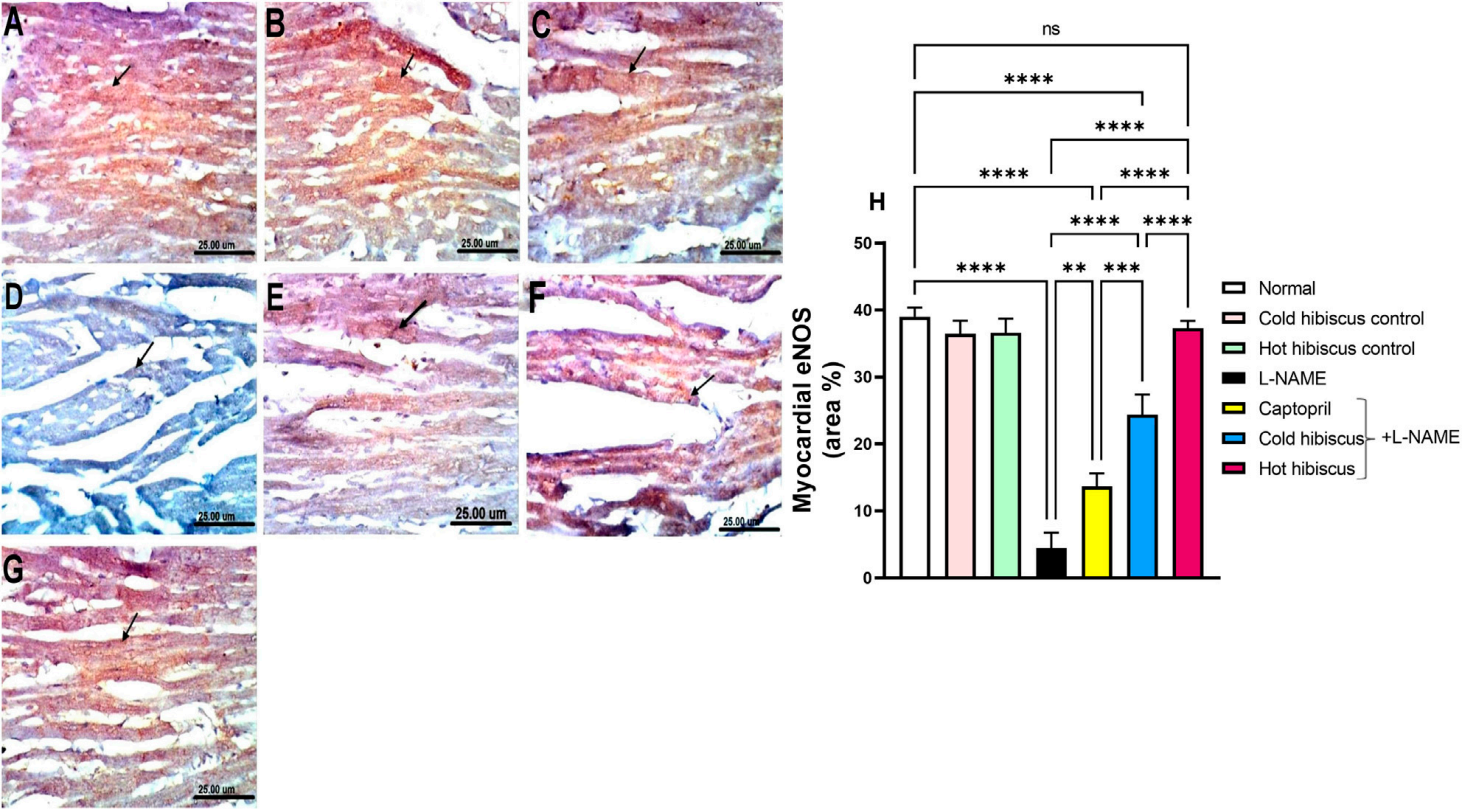
Effect of cold and hot *Hibiscus* extracts on myocardial eNOS immunostaining in L-NAME–induced hypertensive rats. Immunohistochemical analysis of eNOS expression in the heart (Scale bar: 25 um): **(A)** normal rat, **(B, C)** cold *Hibiscus* and hot *Hibiscu*s control rats, respectively, showing intense eNOS expression **(D)** L-NAME-treated rats showing no eNOS expression(arrow) **(E)** Captopril +L-NAME-treated rats showing weak eNOS expression (arrow). **(F)** cold *Hibiscus* + L-NAME-treated rats showing moderate eNOS expression (arrow) **(G)** hot *Hibiscus*+ L-NAME-treated rats showing intense eNOS expression. **(H)** eNOS immunohistochemical staining area %. Results were expressed as means ± SEM (*n* = 5). Statistical analysis was conducted using one-way ANOVA followed by Tukey’s *post hoc* test *p < 0.05, **p < 0.01, ***p < 0.01, ****p < 0.0001, ns: no significance.

**FIGURE 8:**
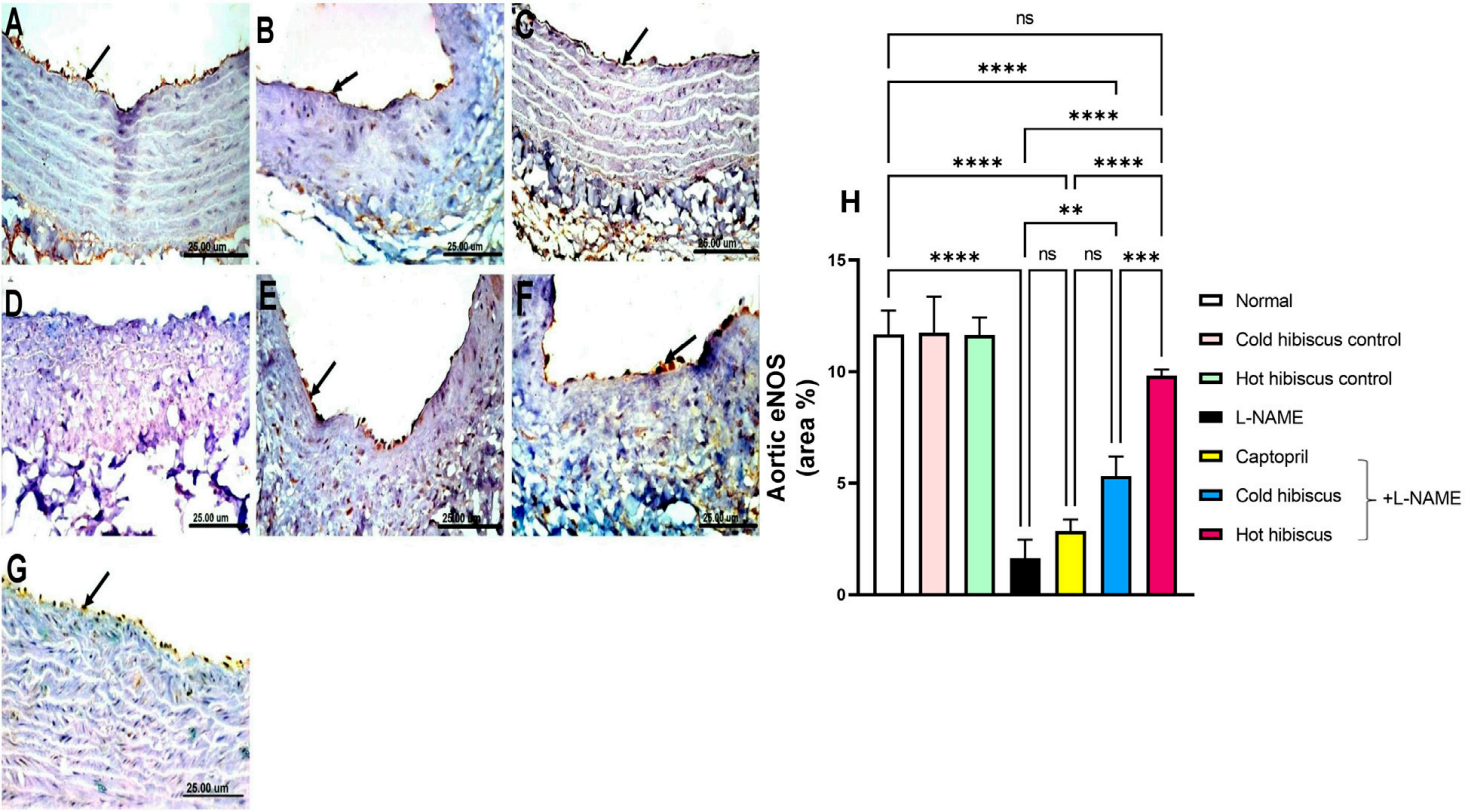
Effect of cold and hot *Hibiscus* extracts on aortic eNOS immunostaining in L-NAME–induced hypertensive rats. Immunohistochemical analysis of eNOS expression in the endothelial cells of aorta (Scale bar:25 um): **(A)** normal rat, **(B,C)** cold *Hibiscus* and hot *Hibiscus* control rats, respectively, showing intense eNOS expression. **(D)** L-NAME–treated rats showing no eNOS expression(arrow) **(E)** Captopril +L-NAME-treated showing weak eNOS expression (arrow). **(F)** cold *Hibiscus* + L-NAME-treated rats showing moderate weak eNOS expression (arrow) **(G)** hot *Hibiscus*+ L-NAME-treated rats showing intense eNOS expression. **(H)** eNOS immunohistochemical staining area %. Results were expressed as means ± SEM (*n* = 5). Statistical analysis was performed using one-way ANOVA followed by Tukey’s *post hoc* test *p < 0.05, **p < 0.01, ***p < 0.01, ****p < 0.0001, ns: no significance.

eNOS immunostaining in the heart and aorta revealed intense expressions in the sections of normal, cold, and hot *Hibiscus* groups (Figure 7A–C, Figure 8A–C). On the contrary, weak or no positive immune reaction was observed in cardiomyocytes and the aorta of rats treated with L-NAME (Figure 7D, Figure 8D). However, sections from rats treated with captopril + L-NAME revealed a weak eNOS expression (Figure 7E, Figure 8E). Meanwhile, a moderate eNOS immune reaction was recorded in sections of rats treated with cold *Hibiscus* + L-NAME (Figure 7F, Figure 8F). An intense eNOS expression appeared in heart and aorta sections of rats treated with the hot *Hibiscus* extract (Figure 7G, Figure 8G). These results were further affirmed with the quantitative estimation of cardiac and aortic eNOS immunostaining in different groups, showing a significant difference between the two extracts for the favor of the hot extract (Figure 7H, Figure 8H).

#### Effect of Cold and Hot *Hibiscus* Extracts on the Plasma Nitric Oxide Level in L-NAME–Induced Hypertensive Rats

**FIGURE 9:**
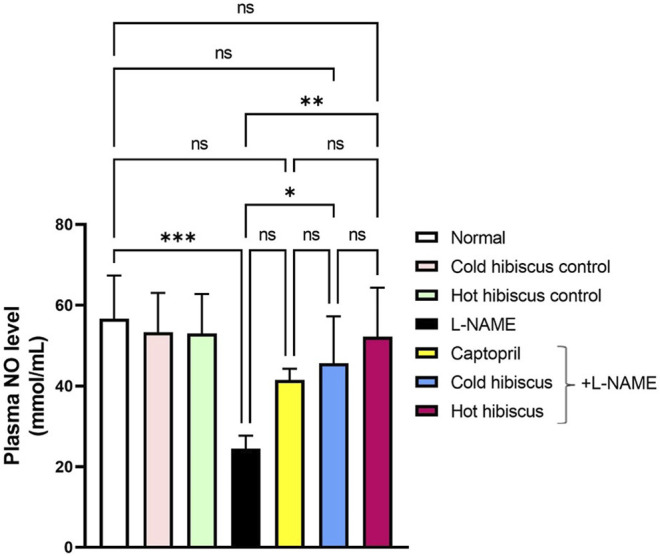
Effect of cold and hot *Hibiscus* on plasma NO levels in L-NAME–induced hypertensive rats. Hypertension was induced by oral administration of L-NAME (40 mg/kg/day) for 4 weeks. Captopril (30 mg/kg) and cold or hot *Hibiscus* (250 mg/kg/day) were given orally for further 4 weeks during which L-NAME administration was continued. Results were expressed as mean ± SEM (*n* = 5). Statistical analysis was performed using one-way ANOVA followed by Tukey’s *post hoc* test. *p < 0.05, **p < 0.01, ***p < 0.001, ****p < 0.0001, ns: no significance.

The plasma NO level was significantly diminished in the hypertensive group when compared to normal animals. However, treatment with cold and hot *Hibiscus* extracts boosted the NO level to nearly approach the normal level but with a higher value displayed with the hot extract (**p < 0.01) (Figure 9). Thus, the hot *Hibiscus* extract is more effective in increasing the NO level and subsequently more efficient in lowering the elevated BP.

### Metabolic Profiling of Hot and Cold *Hibiscus* Extracts by UPLC–MS/MS Analysis

**FIGURE 10:**
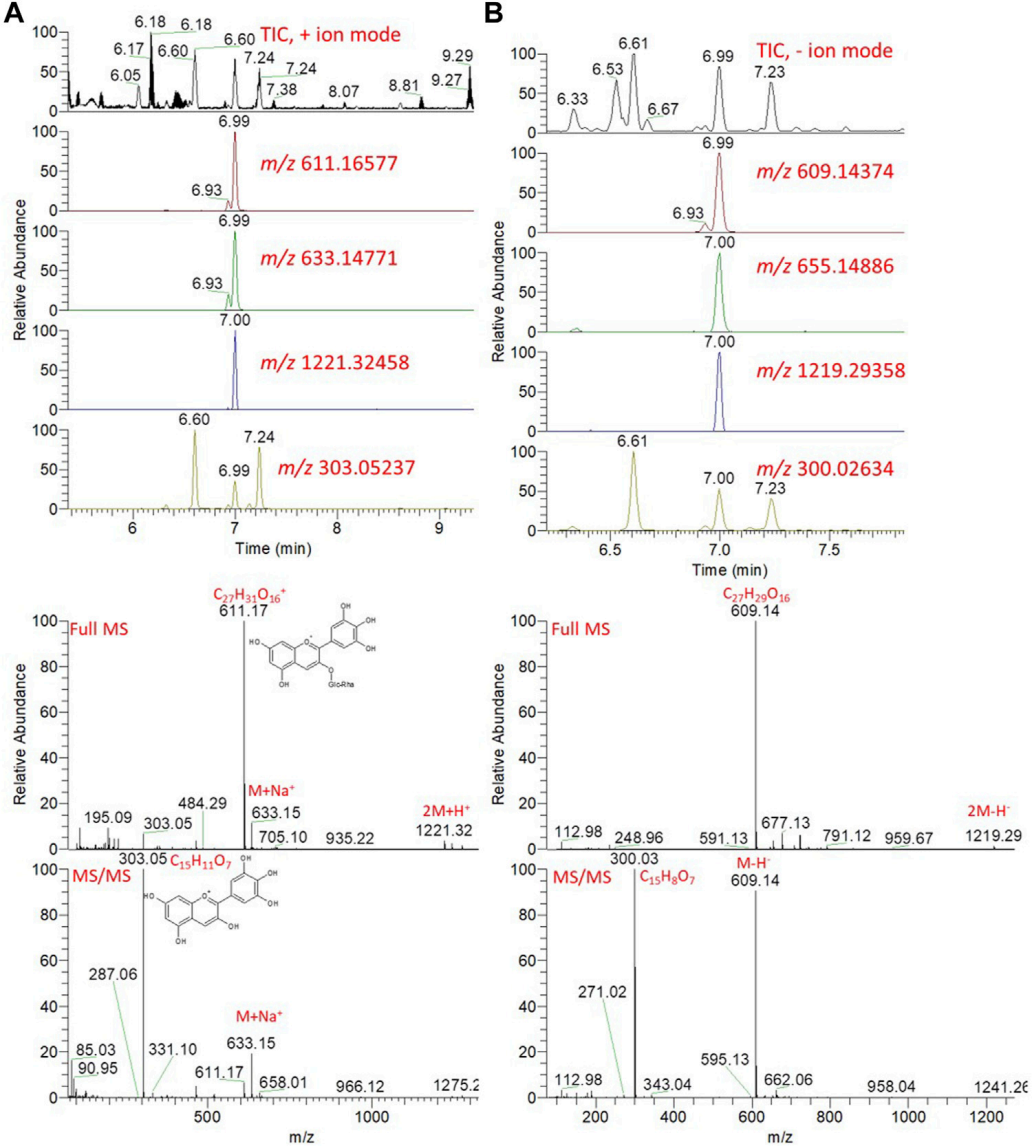
Total ion chromatogram (TIC) and extracted ion chromatograms (EIC) of the peak at m/z representing the anthocyanin, delphinidin-3-neohesperidoside, measured by UPLC/MS positive **(A)** and negative **(B)** ionization mode. Full MS and MS/MS spectra are shown.

Comprehensive chemical profiles of *Hibiscus* aqueous extracts, in response to cold maceration and hot decoction, were obtained by UPLC–MS/MS analysis (Supplementary Figure S2). The separation methods, which were conducted on a reversed-phase LC column, allowed the elution of the metabolites in order of increasing hydrophobicity. For instance, amino acids were eluted first followed by organic acids, whereas flavonoids, glycosides, and anthocyanins were the last to be eluted. Metabolites were annotated based on the comparison of the retention times and mass spectra (monoistopic masses, isotope distribution, and fragmentation patterns) with an in-house spectral library, as well as the previously published *Hibiscus* metabolomics data (Rasheed et al., 2018; El-Shiekh et al., 2020; Salem et al., 2020). An exemplary description for the annotation of metabolites is described in Figure 10 and Supplementary Figures S3, S4.

*Hibiscus* calyces are rich in anthocyanins, including delphinidin and cyanidin, which were glycosylated by the sugar moieties of hexoses and deoxy hexoses. Since these molecules are positively charged, the molecular ions can be easily detected, although they can be also nicely detected in the negative ionization mode. The detailed putative identification of delphinidin-3-neohesperidoside is shown in Figure 10 and Supplementary Figure S3. The extracted ion chromatograms (EICs) of the protonated compound at *m/z* 611.16577 [M]^+^ and a retention time of 6.99 min were detected (Figure 10). The predicted molecular formula for this adduct was C_27_H_31_O_16_^+^. The MS spectra of the compound showed the sodiated adduct [M + Na]^+^ and a dimer of the protonated adduct [2M + H]^+^ at *m/z* 633.14771 and 1221.32458, respectively. In the negative ionization mode, a deprotonated adduct at *m/z* 609.14374 and deprotonation followed by the addition of formic acid [M + FA-H]^-^ at *m/z* 655.14886 were detected at the same RT. A dimer of the deprotonated adduct [2M-H]^-^ at *m/z* 1219.29358 was also detected. In the MS/MS spectra, a peak for a protonated fragment after the loss of the deoxy-hexose moiety [M-rhamnosyl + H]^+^ was detected at *m/z* 465.10715 (M-146.06). Among the detected fragments, the most abundant was the ion at *m/z* 303, corresponding to the loss of the hexoside sugar moiety (Δ162 amu), after the loss of the deoxy-hexose moiety. These fragments were nicely co-eluted as their precursor adduct at the same retention time. The delphinidin group was confirmed by *m/z* 303, representing the loss of the disaccharide moiety *via* cleavage of the glycosidic bondage [M-rhamnosylglucosyl + H]^+^ (Supplementary Figure S3).

The total ion chromatogram revealed that the compound that appeared at the retention time (Rt) 4.3 min was proposed to be caffeoylquinic acid (phenolic acid), which showed molecular ion peaks at m/z 355.10492 and 353.08586 using positive and negative ionization modes, respectively (Supplementary Figure S4). Moreover, the fragmentation pattern resulted from MS/MS showed that the presence of a peak at *m/z* 179.03 represents [caffeoyl-H]-, at m/z 135.04 represents [caffeoyl-H-CO_2_]-, at m/z 191.05 represents [quinic acid-H]-, and at m/z 353.0858 represents [M-H]^-^ (Ruan et al., 2019). The analysis revealed the presence of different classes of metabolites, including sugars, organic and phenolic acids, flavonoids, and anthocyanins.

**FIGURE 11:**
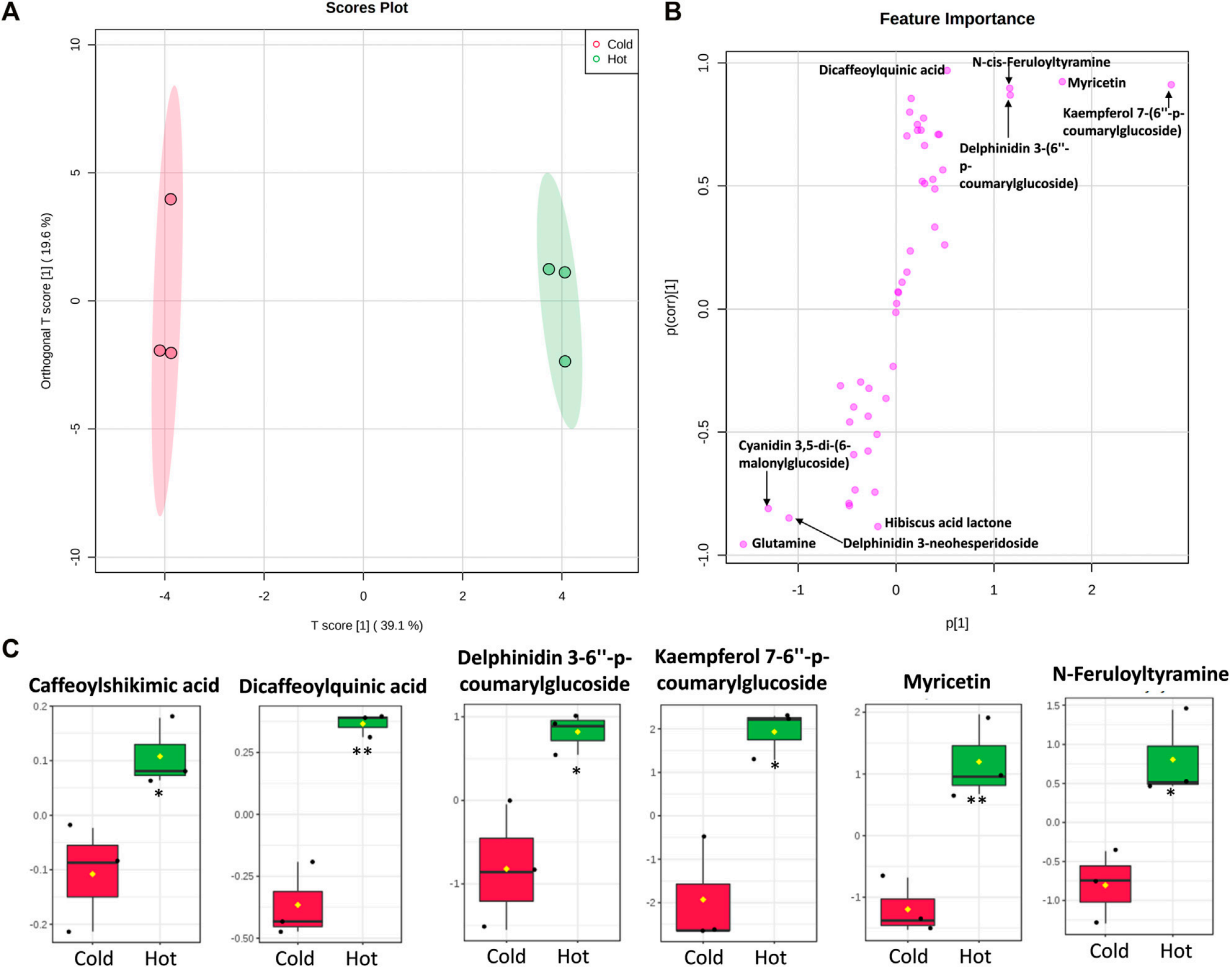
Multivariate data analysis (MVDA) of metabolites identified from *Hibiscus* cold and hot extract. Score plots of OPLS-DA **(A)** and S-plot of OPLS-DA **(B)** based on the UPLC/MS data. Metabolites **(C)** that were significantly higher in *Hibiscus* hot extracts. Log_2_ fold change of metabolite abundance has been used for the boxplots. *Significant difference at *p* < 0.05, **Significant difference at *p* < 0.01.

To characterize the metabolite differences between cold and hot *Hibiscus* extraction, multivariate data analysis was employed. The unsupervised principal component analysis (PCA) revealed two different clusters for the cold and hot extracts along the PC1, which explained 54% of the total variance (Supplementary Figure S5). Orthogonal projections to latent structures discriminant analysis (OPLS-DA) is a supervised method that has great potentiality to identify metabolite markers between two groups (Worley and Powers, 2013). OPLS-DA revealed a clear separation between the two extraction methods (Figure 11). The S-plot revealed that flavonoids, anthocyanins, and organic acids were the major contributors for the difference between cold and hot extraction. Certain metabolites were found in high quantities in the cold extract as *Hibiscus* acid lactone, delphinidin 3-neohespridoside, glutamine, and cyaniding 3,5-di-6-malonylglucoside, while other metabolites including *N*-Feruloyltyramine, caffeoylshikimic acid, dicaffeoylquinic acid, delphinidin-3,6″-p-coumarylglucoside, kaempferol-7,6″-*p*-coumarylglucoside, and myricetin were found in major quantities in the hot extract (Figure 11 and Supplementary Figure S6, S7).

## Discussion

Hypertension is a major chronic disease defined with high arterial blood pressure that affects many people all over the world (Gaziano et al., 2010; Sanchis-Gomar et al., 2016). Uncontrolled hypertension predisposes the patient to many critical diseases such as coronary heart diseases, kidney dysfunction, atherosclerosis, and cerebrovascular complications (Kahan, 2014; Staessen, 2014). Therefore, optimum treatment of hypertension could strongly prevent premature mortality. However, it is not always feasible to achieve the target blood pressure clinically using monotherapy, especially if other co-morbidities are found. Thus, prescribing multi-antihypertensive drugs would subject the patients to several factors that threaten the efficacy of the antihypertensive therapies, including occurrence of drug-induced adverse effects, poor patient compliance, and high economic burden (Pruijm et al., 2008). Hence, herbal remedy is now being considered a good adjunct in managing hypertension and its complications due to its lesser side effects and affordable price that leads the way for research to extract new drugs of natural origin (Obouayeba et al., 2014). Among the most traditionally used antihypertensive and cardioprotective medicinal herbs is *Hibiscus sabdariffa* L. which is known as Karkadeh (Arabic) and Roselle (English), according to the geographic region (Da-Costa-Rocha et al., 2014). It is an annual crop which is cultivated in tropical and subtropical regions (Mahadevan et al., 2009). Egypt is considered among the main *Hibiscus*-producing countries along with China, Mexico, and Thailand (Ramirez-Rodrigues et al., 2011). The calyx is the most commercial part of the plant which can be soaked in water to provide cold or hot colorful herbal drinks and juices in many countries (Mohamed et al., 2013). The *Hibiscus* calyx juice is a healthy drink that has high contents of anthocyanins, vitamin C, and antioxidants (Da-Costa-Rocha et al., 2014). *Hibiscus* is known for its pharmacological properties, including antihypertensive, antioxidant, diuretic, and nephroprotective properties, and many other beneficial effects.

Our previous investigations showed that *Hibiscus* aqueous extracts, among eight extraction solvents, showed promising *in vitro* ACE inhibition capacity (Salem et al., 2020), which prompted us to evaluate the *in vivo* antihypertensive potential of the aqueous extract with respect to its metabolic profile. This study aimed to specify the best method for extraction of the antihypertensive metabolites of *Hibiscus sabdariffa* L. calyces and compare the beneficial effect of both hot and cold aqueous extracts on the blood pressure and heart through two main mechanisms: (1) the vasodilation mediated *via* eNOS/iNOS/NO pathway and (2) the effect on the renin angiotensin aldosterone system (RAAS) through ACE inhibition. The experimental hypertensive model that mimics hypertension to a great extent in humans is the L-NAME–induced hypertension model (Ramanathan and Thekkumalai, 2014). Accordingly, it was chosen to conduct our experiment due to its validity and well-establishment. In the current study, L-NAME induced a significant increment in blood pressure; however, it displayed changes in ECG parameters but did not reach significance. Previous studies showed non-significant changes in ECG on using L-NAME and linked the elevated R-R interval to the decrease in the heart rate (Conceição-Vertamatti et al., 2020). The prolongation of the QTc interval is considered an indicator of the changes in ventricular performance, as denoted in previous studies (Fish Jeffrey et al., 2004; Mozos and Caraba, 2015). Nonetheless, the discrepancy in the ECG changes encountered on administration of L-NAME could be due to the difference in species and age of the rats, along with the dose and duration of L-NAME. Considering the impact on vasodilation, administration of L-NAME and its conversion into the active N(omega)-nitro-L-arginine (L-NOARG) in turn inhibited eNOS, leading to the deficiency of the potent vasodilating NO and subsequently the occurrence of systemic vasoconstriction and hypertension (Pfeiffer et al., 1996; Hopkins et al., 2013). This was shown by the elevated blood pressure in the current study and the histological derangement seen in the aorta and the myocardium. These results are in line with the previous studies that showed elevated blood pressure on using L-NAME (Cardoso et al., 2014; Jaarin et al., 2015). Moreover, aortic and cardiac eNOS were significantly reduced, leading to a marked inhibition in the plasma NO, while aortic and myocardial immunohistochemical samples showed an increment in iNOS. Inducible NOS is reported to have a crucial inflammatory role where it is induced inside the macrophages, leading to tissue damage and inflammation (Ogaly et al., 2015). This was displayed histologically in our current study by the presence of the inflammatory cell infiltrate in the aorta and heart, as well as the appearance of focal necrosis of cardiomyocytes associated with inter-myocardial edema. A previous study demonstrated significant reduction in eNOS immunostaining in the heart and aorta of the L-NAME group, as well as the enhanced cardiac and aortic iNOS protein expression, results that are in agreement with our work (Abdel-Rahman et al., 2017). The imbalance in the expression of eNOS and iNOS contributes to the pathogenesis of hypertension where eNOS serves as the main producer of NO that regulates the vascular tone (Abdel-Rahman et al., 2017). By contrast, NO formed by iNOS has a completely different role through causing endothelial impairment *via* the reaction with superoxide radicals and promoting oxidative stress (Oliveira-Paula et al., 2014). Furthermore, iNOS promotes the arginase activity which is known as an opponent to eNOS for L-arginine (source of NO production) (Oliveira-Paula et al., 2014).

Administration of *Hibiscus* extracts slightly reversed the non-significant ECG changes induced by L-NAME. In addition, both *Hibiscus* extracts restored the changes in the immunohistochemical expressions of eNOS and iNOS in the heart and aorta that was reflected on boosting the plasma NO level and regression of the histological damage of aortic and heart tissues. However, superior results were shown on administering the hot extract. Ajay et al. (2007) and Lim et el., (2017) described the hypotensive effect of the *Hibiscus* extract through potentiating the endothelium-derived NO-cGMP (cyclic guanosine monophosphate) pathway and preventing the Ca^2+^ influx into the vascular tissues (Ajay et al., 2007; Lim et al., 2017). Another possible mechanism that explains the vasodilatory effect of the *Hibiscus* extract is activation of phosphatidylinositol 3-kinase (PI3K)/protein kinase B (AKT) signaling that phosphorylates eNOS, rendering it active (Sarr et al., 2009).

Concerning the RAAS arm, L-NAME administration showed activation in RAAS, as evidenced by the rise in plasma ACE, angiotensin II, and aldosterone levels. Seth et al. (2016) demonstrated the elevated level of the ACE on using L-NAME, suggesting RAAS as an advocate to L-NAME–induced hypertension (Seth et al., 2016). Herein, the justification could be due to the diminished NO production on L-NAME dosing, as reported in the current study, which in turn manifests vasoconstriction that leads to low renal perfusion. Consequently, the kidney releases renin from the macula densa of the juxtaglomerular (JG) cells. In the circulation, renin will convert angiotensinogen to angiotensin I. Here comes the role of the ACE to convert angiotensin I (inactive) to angiotensin II (active) which is a potent vasoconstrictor. Moreover, angiotensin II stimulates the adrenal production of aldosterone, which augments distal tubular sodium and water reabsorption, contributing to aggravation of hypertension.

*Hibiscus* hot and cold extracts dampened the elevated levels of the plasma ACE, angiotensin II, and aldosterone activities *in vivo*, as previously reported (Nurfaradilla et al., 2019). Previous studies linked the inhibition of the ACE to the phenolic and anthocyanin fractions (delphinidin-3-*O*-sambubiosides and cyanidin-3-*O*-sambubiosides) that compete with the active enzymatic site, leading to its inhibition, and consequently lowering the level of angiotensin II and secretion of aldosterone, thus revealing its ACE inhibition and diuretic potentials (Ojeda et al., 2010; Salem et al., 2020). These effects were confirmed by the *in vitro* ACE and renin inhibition assays demonstrating that both hot and cold *Hibiscus* extracts inhibited the ACE and renin enzymes. Although the hot extract showed a slightly higher ACE inhibition activity than the cold extract, both extracts showed similar inhibition activity towards renin. Intriguingly, the level of *Hibiscus* major anthocyanins such as delphinidin-3-*O*-sambubiosides and cyanidin-3-*O*-sambubiosides tend, although non-significant, to be higher in the hot extracts, indicating the contribution of other metabolites to the observed potentiality. These ACE and renin inhibition assays contribute to the antihypertensive effects of *Hibiscus sabdariffa*.

Tracing specific metabolites responsible for the antihypertensive activity for both hot and cold extracts using UPLC–MS/MS analysis illustrated the presence of anthocyanins and phenolic compounds in both hot and cold extracts. Equivalent anthocyanin concentration was obtained by cold or hot extraction, while the total phenolics were shown to be better extracted with hot water (Ramirez-Rodrigues et al., 2011). These results aided to interpret the superior antihypertensive effects that were uncovered on using the hot *Hibiscus* extract since the higher concentration of total phenolics added an extra benefit to the hot extract relative to the cold one. As we mentioned before, several studies linked the hypotensive property of *Hibiscus sabdariffa* to its anthocyanin content (Herrera-Arellano et al., 2007; Gurrola-Diaz et al., 2010; Mckay et al., 2010; Yang et al., 2010). However, the pivotal role of polyphenolic compounds showed their importance in lowering the blood pressure and cholesterol level through restoring the balance between LDL and HDL and declined total cholesterol, thus preventing atherosclerosis and impeding cardiovascular diseases (Carvajal-Zarrabal et al., 2005; Yang et al., 2010).

Administration of hot and cold *Hibiscus* extracts markedly reduced the elevated blood pressure that could be related to the anthocyanins’ hypotensive constituent of *Hibiscus sabdariffa*, including delphinidin-3-*O*-sambubioside (hibiscin) and cyanidin-3-*O*-sambubioside (gossypicyanin), which were reported to possess the ACE inhibition activity (Ojeda et al., 2010). Both metabolites showed higher accumulation, still non-significant, in the hot extract. The superiority of the hot extract could be related to the efficient extraction of phenolic acids, especially caffeic acid derivatives such as dicaffeoylquinic acid and caffeoylshikimic acid. Dicaffeoylquinic acid exerted a moderate antihypertensive effect in spontaneously hypertensive rats (Simeonova et al., 2016). Caffeic acid injection caused the SBP to reduce anesthetized spontaneously hypertensive rats (SHR) (Zhao et al., 2012). The highest level of *N*-feruloyltyramine (NFT) was observed in the hot extract. The pharmacological effects of *Smilax aristolochiifolia* against hypertension in mice were attributed mainly to the presence of NFT because the fractions possessed the major effects which were those contained in higher concentrations of this compound (Amaro et al., 2014). Moreover, myricetin, which was detected in higher levels in hot extracts, possessed both *in vivo* and *in vitro* antihypertensive activities, especially through reducing the oxidative stress, in addition to its potential to affect the heart rate, vascular reactivity, and systolic blood pressure (Borde et al., 2011). The levels of a *p*-coumaroylated flavonoid and *p*-coumaroylated anthocyanin, namely, kaempferol 7-(6″-*p*-coumarylglucoside) and delphinidin 3-(6″-*p*-coumarylglucoside), were detected at higher levels in the hot extract. The phenolic content presented promising antihypertensive activity (Liu et al., 2012; Shen et al., 2019).

## Conclusion

*Hibiscus sabdariffa* represents a traditionally used antihypertensive herb. The hot aqueous extract of *Hibiscus sabdariffa* recorded the best activity in lowering the high blood pressure and restoring the normal histology of the heart compared to the cold extract and captopril. Moreover, the *in vivo* studies illustrated that the hot *Hibiscus* extract produced its antihypertensive effect through reducing the plasma ACE, angiotensin II, and aldosterone levels. It also raised aorta and myocardial eNOS and nitric oxide values but reduced the iNOS value, consequently unraveling vasodilation and reducing the blood pressure. The hot extract also amended the histological changes of the myocytes such as necrosis, inflammation, and vacuolation that resulted from using L-NAME. The superiority of the hot extraction method was explained through the UPLC–MS/MS analysis that traced certain metabolites which are implicated in the antihypertensive effect of the extract. Some metabolites were found in high quantities in the hot extract including N-feruloyltyramine, caffeoylshikimic acid, dicaffeoylquinic acid, delphinidin-3,6″-*p*-coumarylglucoside, kaempferol-7,6″-*p*- coumarylglucoside, and myricetin. Therefore, the mystery behind the antihypertensive effects of cold and hot extraction is now partially unraveled where the hot extraction has hypotensive properties and is completely far from the myth of elevating the blood pressure.

## Data Availability

The original contributions presented in the study are included in the article/Supplementary Material, further inquiries can be directed to the corresponding authors.

## Abbreviations

ACC, The American College of Cardiology; ACE, angiotensin-converting enzyme; BP, blood pressure; ECG, electrocardiogram; EIC, extracted ion chromatograms; eNOS, endothelial nitric oxide synthase; iNOS, inducible nitric oxide synthase; HESI, heated electrospray ionization; HHL, Histidine-L-hippuryl- L-leucine-chloride; HRMS, high-resolution mass spectrometry; HSS, high-strength silica; JG, juxtaglomerular; LC, liquid chromatography; L-NAME, L-Nitro-Arginine Methyl Ester; L-NOARG, N omega-nitro-L-arginine; MVDA, multivariate data analysis; NO, nitric oxide; OPLS-DA, Orthogonal Projections to Latent Structures Discriminant Analysis; PCA, principal component analysis; PBS, phosphate-buffered saline; PI3K/AKT, phosphatidylinositol 3-kinase/protein kinase B; RAAS, renin–angiotensin–aldosterone system; SBP, systolic blood pressure.
