# Supplementary material for: A Comparative Study of the Antihypertensive and Cardioprotective Potentials of Hot and Cold Aqueous Extracts of Hibiscus sabdariffa L. in Relation to Their Metabolic Profiles
Source: Front Pharmacol. 2022 Feb 23;13:840478. doi: 10.3389/fphar.2022.840478 (PMC8905494; doi:10.3389/fphar.2022.840478)
Supplement: Supplementary file 1 [file DataSheet1.pdf]

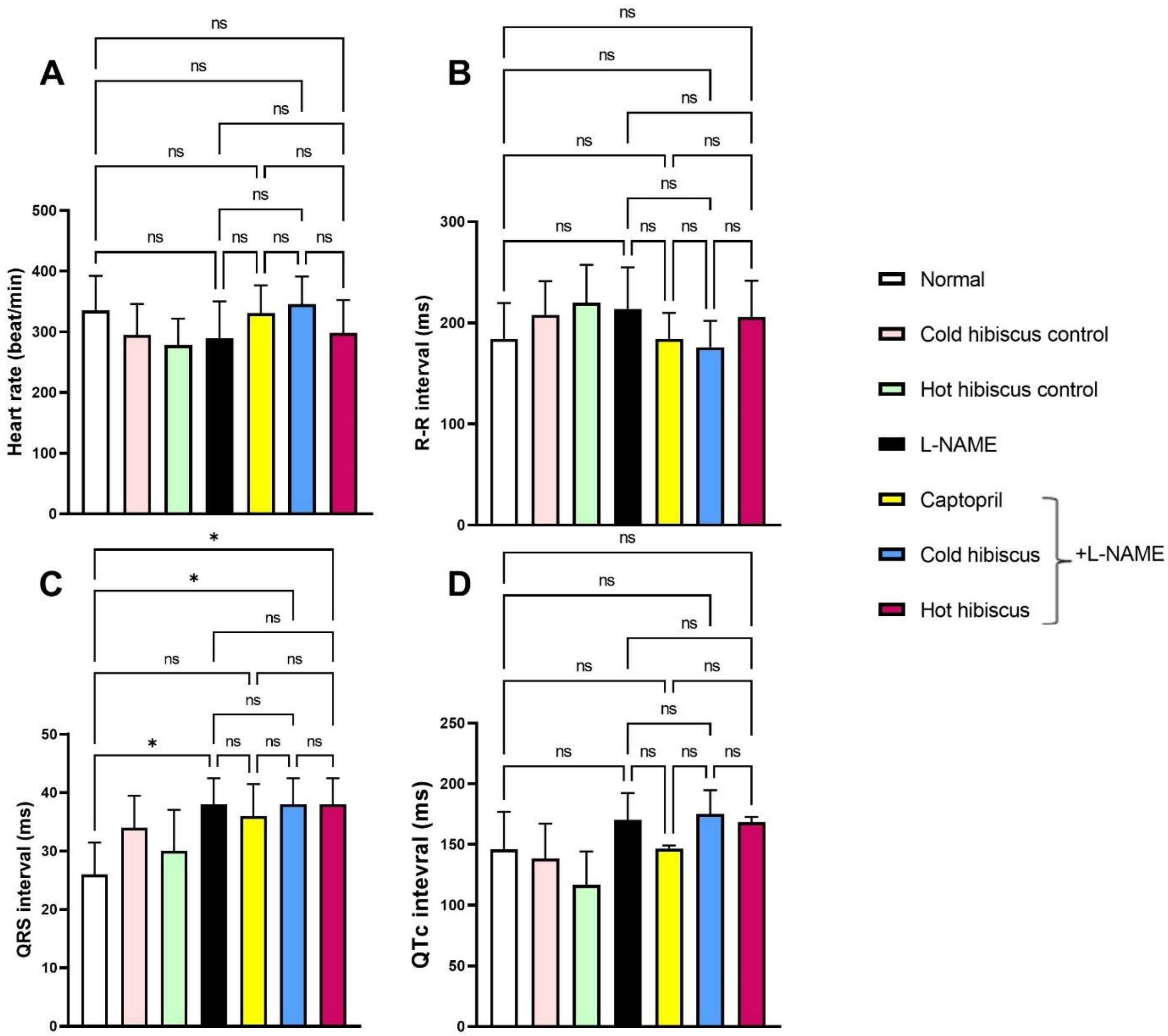


**Figure S1. Effect of cold and hot hibiscus extracts on Heart rate (A), R-R interval (B), QRS interval (C) and QTc interval (D) in L-NAME-induced hypertensive rats**

Hypertension was induced by oral administration of L-NAME (40 mg/kg/day) for 4 weeks. Captopril (30 mg/kg), cold or hot hibiscus extract (250 mg/kg/day) was given orally for further 4-weeks during which L-NAME administration was continued. Results were expressed as means ± SEM (n=5). Statistical analysis was done using One way ANOVA followed by Tukey's post-hoc test. *p < 0.05, **p < 0.01, ***p < 0.001, ****p < 0.0001, ns: no significance.

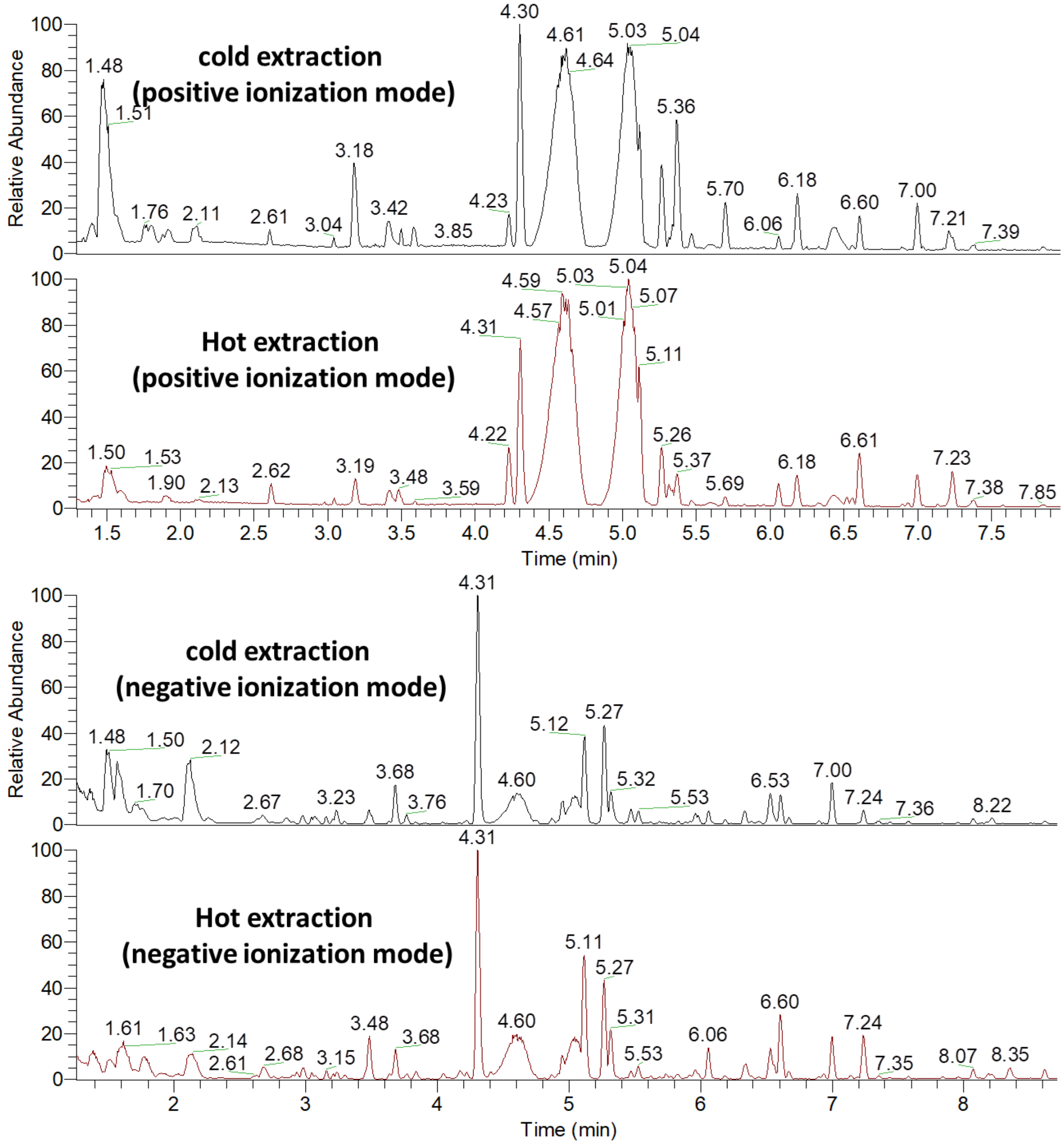


**Figure S2: Total ion chromatogram of hibiscus cold and hot extracts measured in positive and negative ionization mode.**

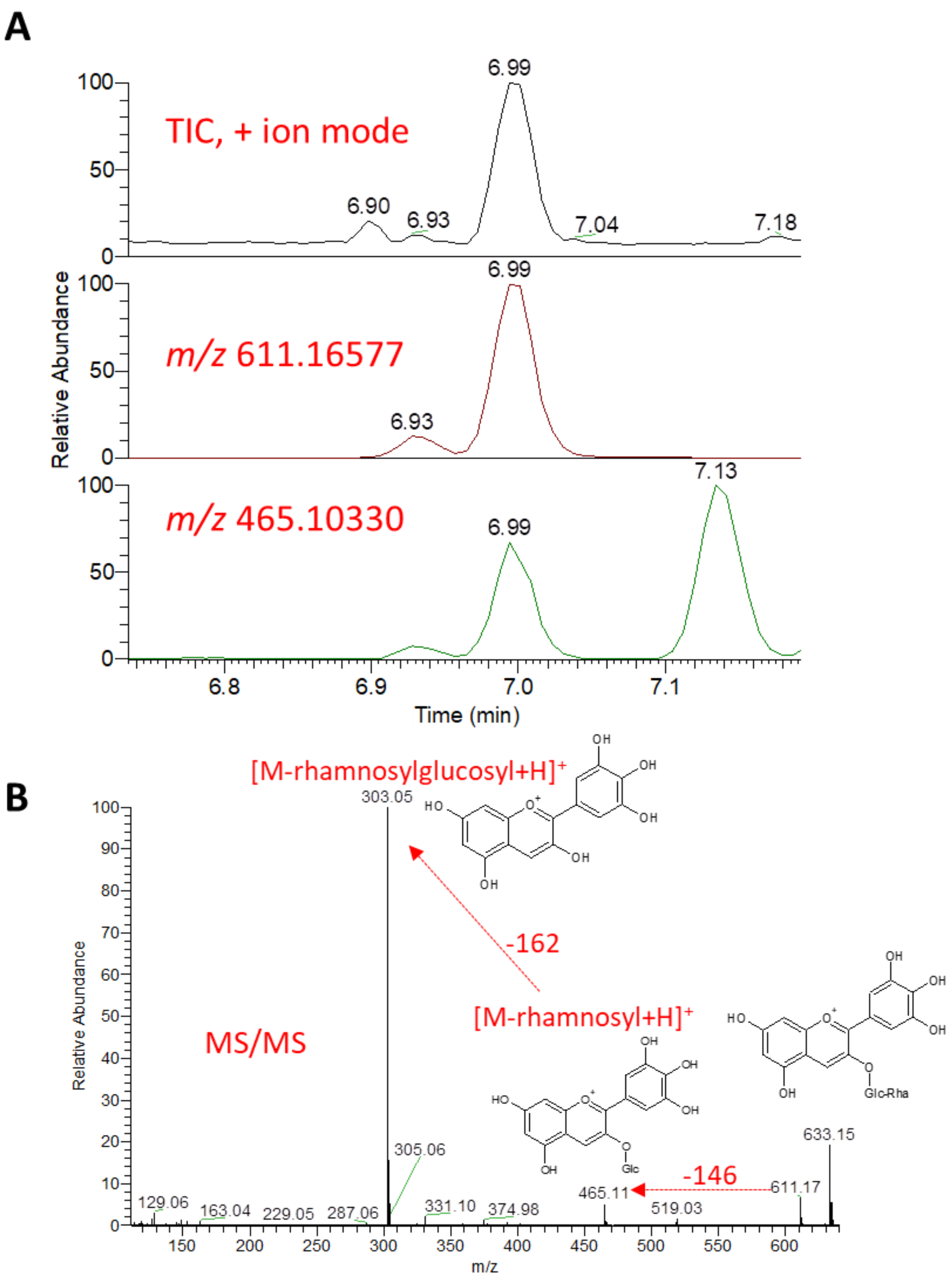


**Figure S3. Identification of delphinidin-3-neohesperidoside based on the fragmentation pattern.**

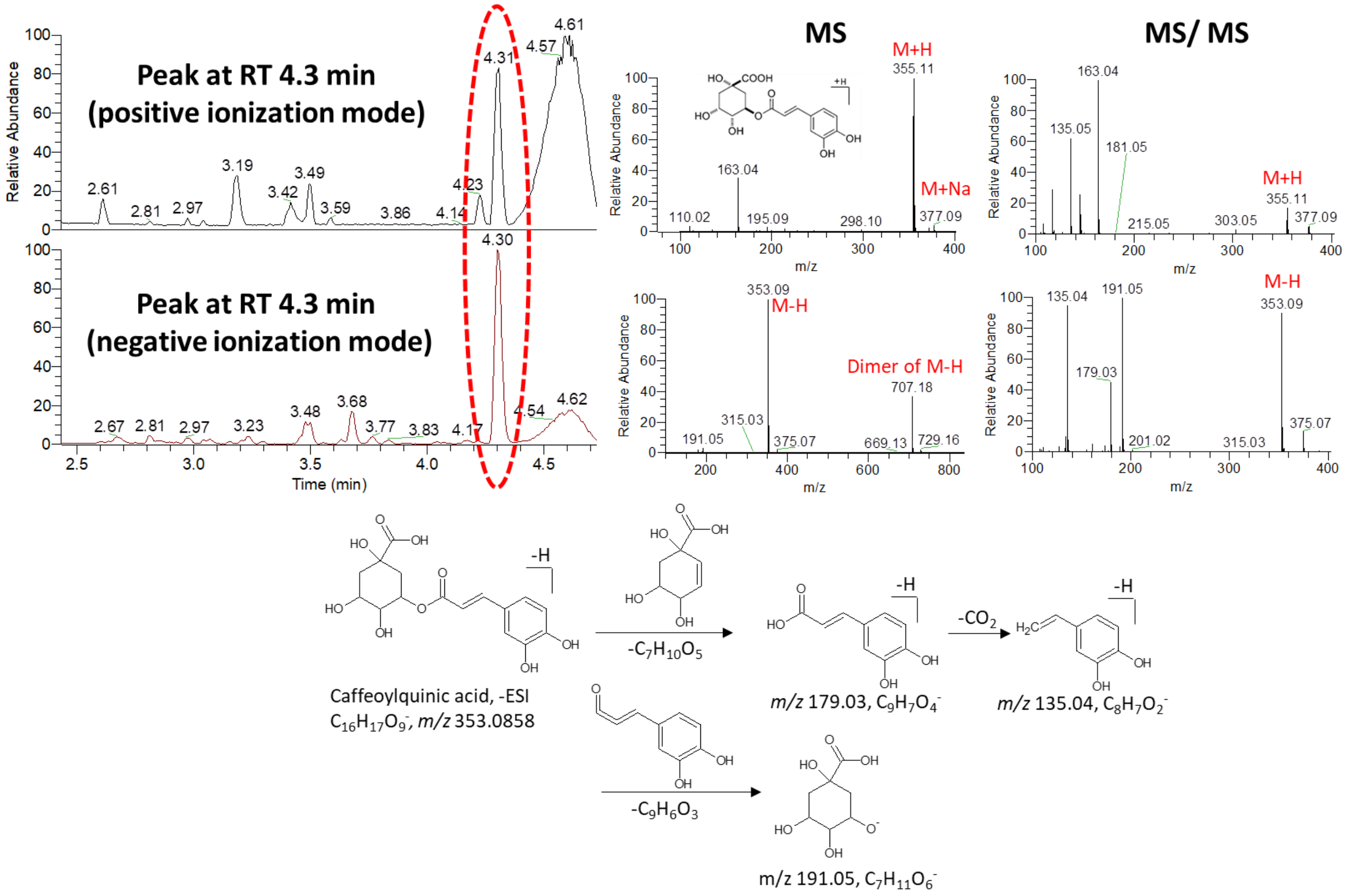


**Figure S4. Representative example for identification of caffeoylquinic acid from Hibiscus extract measured in positive and negative ionization mode.** Schematic diagram showing the production of fragment ions acid during MS/MS analysis is shown. Peak at *m/z* 179.03 represents [caffeoyl-H]$^-$, at *m/z* 135.04 represents [caffeoyl-H-$CO_2$]$^-$ , at *m/z* 191.05 represents [quinic acid-H]$^-$ and at *m/z* 353.0858 represents [M-H]$^-$.

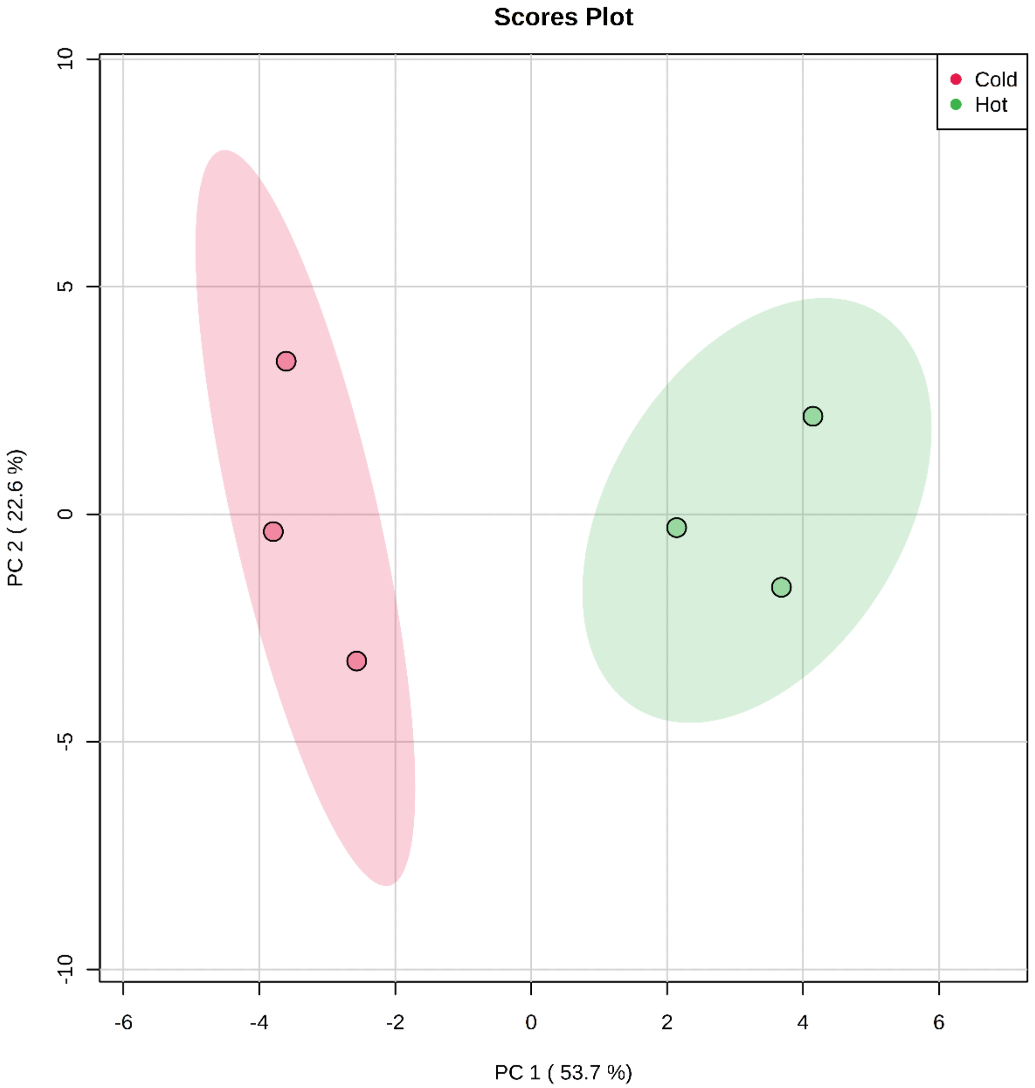


**Figure S5. Principal component analysis (PCA) score plot of metabolites identified from Hibiscus cold and hot extracts**

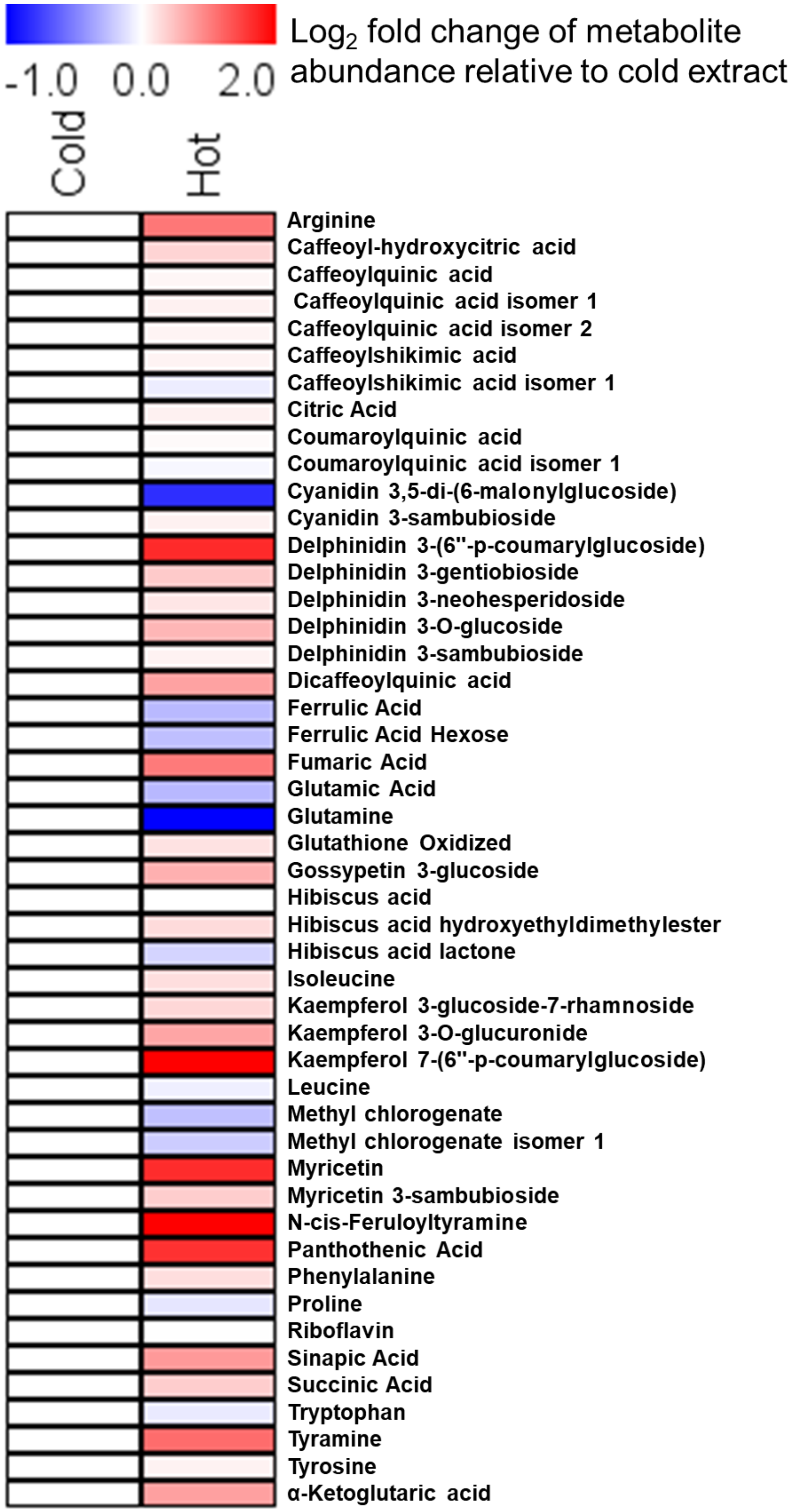


**Figure S6. Heatmap represents the relative abundance of metabolites extracted from cold and hot Hibiscus extracts.**

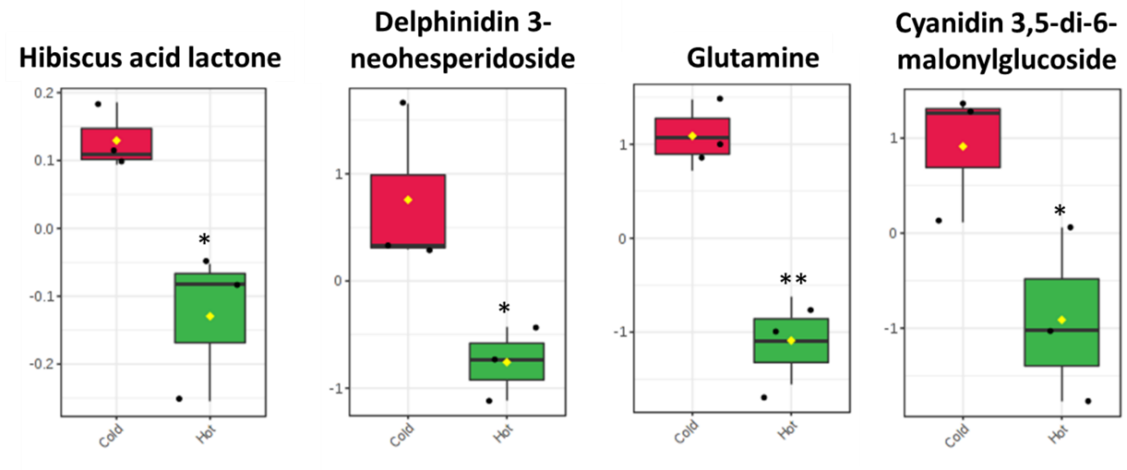


**Figure S7. Metabolites that were significantly higher in Hibiscus cold extract**. $Log_2$ fold change of metabolite abundance has been used for the boxplots. $^{*}$Significant difference at $p < 0.05$, $^{**}$Significant difference at $p < 0.01$.
